# National, regional and provincial prevalence of peripheral artery disease in Chinese adults in 2023: an updated systematic review and modelling study

**DOI:** 10.7189/jogh.16.04155

**Published:** 2026-04-17

**Authors:** Jiali Zhou, Shiyi Shan, Guangdian Shen, Boren Tan, Jiayao Ying, Igor Rudan, Peige Song, Global Health Epidemiology Research Group (GHERG)

**Affiliations:** 1 Center of Clinical Big Data and Analytics of the Second Affiliated Hospital and School of Public Health, Zhejiang University School of Medicine, Hangzhou, Zhejiang, China; 2 Zhejiang Key Laboratory of Intelligent Preventive Medicine, Hangzhou, Zhejiang, China; 3 Sir Run Run Shaw Hospital, Zhejiang University School of Medicine, Hangzhou, Zhejiang, China; 4 Department of Cardiology, The Second Affiliated Hospital of Zhejiang University School of Medicine, Hangzhou, Zhejiang, China; 5 Nuffield Department of Primary Care Health Sciences, University of Oxford, Oxford, UK; 6 Centre for Global Health, Usher Institute, University of Edinburgh, Edinburgh, Scotland, UK

## Abstract

**Background:**

Peripheral artery disease (PAD), a common atherosclerotic condition characterised by the narrowing or obstruction of peripheral arteries, has become a substantial public health concern in China. However, its prevalence across the country at the provincial level has never been quantified. Through an updated systematic review and modelling study, we aimed to estimate the national, regional, and provincial prevalence of PAD among Chinese individuals aged 30–89 years in 2023.

**Methods:**

We conducted an updated literature search in PubMed, Embase, MEDLINE, the China National Knowledge Infrastructure, Wanfang, and the Chinese Science and Technology Journal Database to identify studies published between 4 March 2017 and 12 August 2024 reporting on the prevalence of PAD in the general Chinese population. We also identified and included studies from previous systematic reviews. We then used a multilevel mixed-effects meta-regression model to generate the age- and sex-specific prevalence estimates of PAD among individuals aged 30–89 years at the national level. We calculated pooled odds ratios (ORs) for factors associated with PAD using a random-effects meta-analysis and incorporated them into an ‘associated factor-based model’ to estimate regional and provincial prevalence.

**Results:**

We included 54 articles from all searches. Model-based estimates indicated that the overall prevalence of PAD among Chinese adults aged 30–89 years in 2023 was 5.47% (95% confidence interval (CI) = 4.64–6.44), equivalent to 49.90 million (95% CI = 42.32–58.73) affected individuals. Prevalence increased progressively with age, ranging from 4.23% (95% CI = 3.58–5.00) among individuals aged 30–34 years to 18.37% (95% CI = 15.90–21.13) among those aged 85–89 years. The overall prevalence was higher in females (6.19%; 95% CI = 5.25–7.27) than males (4.76%; 95% CI = 4.03–5.61). Regionally, Northeast China had the highest prevalence of PAD at 5.87% (95% CI = 4.98–6.91), while Central China had the lowest at 5.26% (95% CI = 4.46–6.19). At the provincial level, the prevalence of PAD was the highest in Beijing (6.40%; 95% CI = 5.43–7.53) and lowest in Tibet (4.35%; 95% CI = 3.68–5.12). Female sex, current smoking, hypertension, diabetes, coronary heart disease, and stroke were identified as factors significantly associated with PAD.

**Conclusions:**

We found that PAD is prevalent in China, with pronounced age-related trends, sex differences, and regional disparities. These findings could inform targeted public health initiatives and appropriate resource allocation.

**Registration:**

PROSPERO: CRD420251118923.

Peripheral artery disease (PAD) is a common atherosclerotic condition characterised by the narrowing or obstruction of peripheral arteries, most commonly affecting the lower extremities [1-4]. Its clinical manifestations range from mild symptoms, such as intermittent claudication and ischaemic rest pain, to severe complications, including critical limb ischemia, gangrene, and amputation [1–4]. In its early stages, PAD is often asymptomatic or presents with nonspecific symptoms, leading to delays in diagnosis and under-recognition in clinical practice [2,3,5]. Consequently, the disease frequently progresses to advanced stages, where adverse outcomes such as limb loss and cardiovascular events are more likely to occur [1–4]. Despite its impact on cardiovascular disease, disability, and premature mortality, PAD remains markedly underdiagnosed, under-recognised, and undertreated [5,6].

Typically, PAD is diagnosed using the abnormal ankle-brachial index (ABI), defined as the ratio of the systolic blood pressure at the ankle to the systolic blood pressure in the arm [2,4]. The condition’s global prevalence was estimated to be 5.56% among individuals aged ≥25 years and older in 2015, corresponding to approximately 237 million cases, with nearly 73% occurring in low- and middle-income countries (LMICs) [7]. As a populous LMIC, China bears a considerable share of this burden, with previous estimates placing the prevalence of PAD at 5.64% among those aged ≥25 years, amounting to approximately 54.60 million affected individuals in 2015 [7]. However, the epidemiological situation may have worsened in recent years, driven by rapid population ageing, urbanisation, and the rising prevalence of key risk factors such as diabetes and hypertension [8,9].

Accurate and up-to-date data on the prevalence of PAD are essential to support evidence-based policymaking, resource allocation, and the design of targeted prevention and treatment strategies. Region-specific and province-specific estimates are particularly needed to address geographic disparities in disease burden and healthcare access. However, large-scale population-based PAD surveys using ABI are logistically and financially demanding, especially in less-developed regions where diagnostic infrastructure and trained personnel may be limited. In this context, modelling studies based on available epidemiological data provide a valuable, cost-effective alternative for estimating disease burden at national and subnational levels.

Several new epidemiological studies have emerged since the last estimate of the prevalence of PAD in China was published in 2015, offering improved data coverage and quality. These now make it feasible to generate more comprehensive and up-to-date prevalence estimates, including at the provincial level. Here, we applied a global health metrics modelling framework developed by the Global Health Epidemiology Reference Group (GHERG) to estimate the national and provincial prevalence of PAD in mainland China in 2023 [10,11]. Using standardised case definitions based on an ABI of ≤0.90, we aimed to generate model-based estimates of the national, regional, and, for the first time, provincial prevalence and number of individuals affected by PAD; to examine variations in prevalence by age, sex, and region; and to synthesise evidence on factors associated with PAD.

## METHODS

We preregistered this updated systematic review and modelling analysis in PROSPERO (CRD420251118923) and adhered to the PRISMA and GATHER statements in reporting our findings (Sections 1 and 2 in the **Online Supplementary Document**) [12,13].

### Search strategy and study selection

We first estimated the prevalence of PAD in China through a systematic review and modelling analysis in 2019 [14]. To update these estimates, we systematically searched PubMed, Embase, MEDLINE, the China National Knowledge Infrastructure, Wanfang, and the Chinese Science and Technology Journal Database to identify epidemiological studies published between 4 March 2017 and 12 August 2024 reporting on the prevalence of PAD in the general Chinese population. The search strategy incorporated controlled vocabulary and free-text terms related to peripheral artery disease and prevalence, with the term ‘China’ added in the three English databases (Section 3 in the **Online Supplementary Document**). No language restrictions were applied. We also manually screened the reference lists of eligible studies and relevant reviews to identify additional studies.

After removing duplicates and irrelevant records, two reviewers (GS and BT) independently screened titles and abstracts, followed by full-text reviews to determine final eligibility. Studies were included if they were original epidemiologic investigations conducted among the general Chinese population and reporting on the prevalence of PAD, defined as an ABI value ≤0.90 [2,15]. We excluded studies if they focused on special populations (*e.g.* patients with diabetes or inpatients), did not clearly define PAD measurement methods or diagnostic criteria, or lacked numerical data to estimate prevalence. For multiple publications from the same study, we included the one presenting the most comprehensive and updated results.

### Data extraction and quality assessment

Two reviewers (GS and BT) independently extracted data from eligible studies, which were subsequently verified by a third investigator (JZ). Extracted variables included publication details (title, first author, publication year), study characteristics (year of investigation, study location and its corresponding latitude and longitude, study setting, study design, sampling strategy, ABI measurement, diagnostic approach, case definition), sample characteristics (sample size, age range, mean or median age, female proportion), and prevalence (reported prevalence, number of PAD cases). Whenever available, we also extracted age-, sex- and geographic location-specific prevalence estimates. A subset of articles additionally investigated associations of potential associated factors with PAD; only those reporting adjusted odds ratios (ORs) from multivariable analyses with consistent definitions were included for analysis.

Two reviewers (JZ and GS) independently assessed the methodological quality of all included articles using the Joanna Briggs Institute Critical Appraisal Checklist for Prevalence Studies, which evaluates nine criteria [16]. Each article was assigned a total quality score ranging from zero to nine, with higher scores indicating better methodological quality (Table S2 in the **Online Supplementary Document**). Using an established framework for observational studies (Table S5 in the **Online Supplementary Document**), we graded the credibility of evidence for these factors into five levels: convincing (I), highly suggestive (II), suggestive (III), weak (IV), and non-significant (V) [17,18].

Any disagreements during study selection, data extraction, or quality assessment were resolved through discussion until consensus was reached.

### Statistical analysis

**Figure 1:**
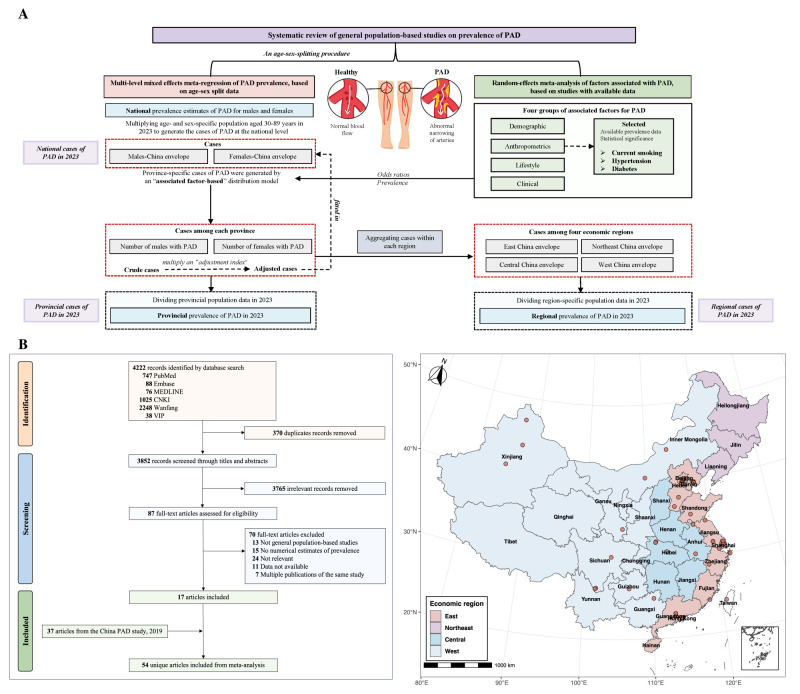
Study flowchart and geographical distribution of included articles. **Panel A.** Study approach for estimating prevalence of PAD at national, regional and provincial levels in 2023. **Panel B.** Study selection and geographical location of the 54 included articles. PAD – peripheral artery disease.

We used the extracted data both for direct evidence synthesis (*i.e.* for calculating the pooled prevalence and meta-analysing ORs) and as inputs for the subsequent multistage modelling analyses (**Figure 1**, Panel A; Section 4 in the **Online Supplementary Document**). We conducted a three-stage modelling analysis to estimate the national, regional, and provincial prevalence of PAD among the general Chinese population in 2023: data preparation, including data imputation and age-sex splitting (stage 1); estimation of national prevalence of PAD in 2023 (stage 2); and estimation of regional and provincial prevalence of PAD in 2023 (stage 3). To ensure reliability and comparability across studies, we restricted the analysis to individuals aged 30–89 years, which encompassed most of the data points available for modelling.

#### Data preparation

For articles that did not report the year of investigation, we imputed the year based on the average lag observed in those that reported both investigation and publication dates (Table S1 in **Online Supplementary Document**). For studies that reported broad geographical units (*e.g.* provinces or multi-city regions), we assigned geographic coordinates based on the mean centre point of the area. Each study location was then categorised into one of four economic regions in mainland China: East China, Northeast China, Central China, and West China (Table S6 in the **Online Supplementary Document**). The study setting (urban, rural, or mixed) was determined according to definitions or descriptions provided in the original articles and classified as ‘mixed’ when not clearly specified or when both urban and rural areas were included without distinction. For studies with censored age bands (*e.g.* ≥70 years), we used two methods to impute the missing age band: if no age statistics were available, the missing band was assumed to have the same width as other age groups in the study; if the average or median age was reported, the missing band was defined by centring it around the reported statistic.

To enhance data availability and comparability for prevalence modelling, we applied an age-sex-splitting procedure to disaggregate aggregated prevalence data into age- and sex-specific estimates (Section 4 in the **Online Supplementary Document**). We employed multilevel mixed-effects meta-regression models to evaluate the association of age and sex with PAD prevalence and to generate prevalence patterns for PAD. Based on the established PAD patterns (Figure S1 in the **Online Supplementary Document**), we further split the PAD prevalence data from contributing articles into sex-specific estimates. Similarly, we divided broad age categories into one-year age intervals based on the fitted age-specific pattern (Figure S1 and Table S3 in the **Online Supplementary Document**).

#### National age- and sex- specific prevalence estimation of peripheral artery disease

To estimate the national prevalence of PAD among individuals aged 30–89 years, we fitted multilevel multivariable mixed-effects meta-regression models based on the aforementioned age-sex split prevalence data, incorporating age and female proportion as fixed effects, with study identification included as a random effect.



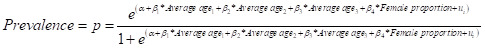



Here, *α* is the intercept term, *β* is the coefficient, and *u_i_* represents the variance of random effects.

Using the above models, we estimated the age- and sex-specific prevalence of PAD at the national level (Table S4 in the **Online Supplementary Document**). We calculated the total number of PAD cases (national envelopes) among Chinese adults aged 30–89 years in 2023 by applying these prevalence rates to the corresponding population data.

Given the observed and anticipated heterogeneity, we used a random-effects model (DerSimonian and Laird method) to calculate pooled prevalence with corresponding 95% confidence intervals (CIs) as part of the sensitivity analysis [19].

#### Regional and provincial age- and sex- specific prevalence estimation of peripheral artery disease

Using a standard approach to translate individual-level risk estimates to the population level, we distributed the estimated national number of PAD cases among Chinese adults aged 30–89 years in 2023 across the 31 provinces in mainland China through an associated factor-based model. This model, initially proposed by GHERG, has been widely applied to estimate disease burden at global, regional, and national levels [7,10]. We then used a random-effects meta-analysis (DerSimonian and Laird method) to synthesise the effects of associated factors with at least three informative data points, including demographic, anthropometric, lifestyle, and clinical factors [19]. Based on data availability and statistical significance, we incorporated three associated factors (current smoking, hypertension, diabetes) into the associated factor-based model. We then estimated the provincial PAD cases (province envelopes) using the following formula:



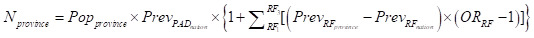



Here, *N_province_* and *Pop_province_* represent the number of PAD cases and population size, respectively, among adults aged 30–89 years in each province or municipality. *Prev_PADnation_* denotes the estimated national prevalence of PAD, while *RF_1_* − *RF_3_* refer to the three selected associated factors (current smoking, hypertension, diabetes). *Prev_RFprovince_* and *Prev_RFnation_* are the prevalence rates of the three associated factors in each province or municipality and at the national level. *OR_RF_* is the synthesised OR of current smoking, hypertension, and diabetes.

To ensure that the sum of provincial cases aligned with the national envelopes, we applied an adjustment applied for each province. The adjusted provincial prevalence of PAD was then calculated by dividing the number of PAD cases in each province or municipality by its corresponding population. Regional PAD cases (regional envelopes) were then developed by aggregating the cases within each economic region (East China, Northeast China, Central China, and West China). The regional prevalence of PAD was calculated as the number of PAD cases in each economic region divided by its respective population.

We performed all analyses in *R*, version 4.4.2 (R Foundation for Statistical Computing, Vienna, Austria), with a two-sided *P*-value <0.05 indicating statistical significance.

## RESULTS

We retrieved 4222 records through database searches, retaining 3852 after deduplication and 87 after title/abstract screening, with 17 meeting the inclusion criteria after a full-text review. We additionally included 37 eligible articles from a previous analysis [14], resulting in a total of 54 articles (**Figure 1**, Panel B; Section 6 in **the** **Online Supplementary Document**). These 54 articles collectively involved 328 969 participants, including 14 714 with PAD. Most articles were published after 2010 (n = 36, 66.67%) and had a sample size greater than 1500 (n = 37, 68.52%). Nearly half of the included articles were conducted in urban settings (n = 23, 42.59%). All included articles received a quality score of five or higher (Figure S2 and Tables S7 and S8 in the **Online Supplementary Document**).

**Figure 2:**
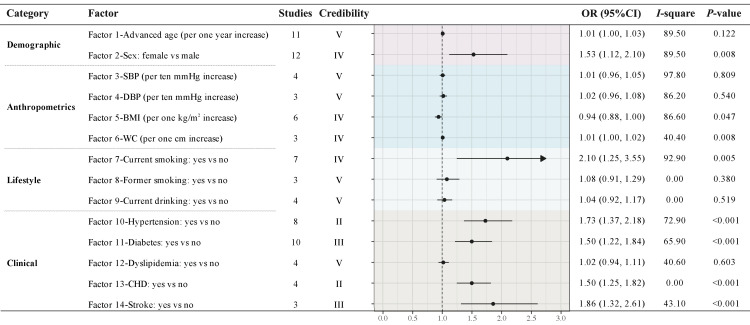
Meta-analyses of associated factors of PAD. Synthesised effect size of 14 associated factors that were investigated in at least three studies using multivariate design. All the factors are classified into four categories: demographic, anthropometrics, lifestyle and clinical factors. Evidence class criteria: Convincing (class I), highly suggestive (class II), suggestive (class III), weak (class IV), and non-significant (class V). BMI – body mass index, CHD – coronary heart disease, CI – confidence interval, DBP – diastolic blood pressure, OR – odds ratio, SBP – systolic blood pressure, WC – waist circumference.

The overall prevalence of PAD among Chinese adults aged 30–89 years was 5.47% (95% CI = 4.64–6.44), corresponding to 49.90 million (95% CI = 42.32–58.73) affected individuals in 2023 (**Figure 2**, Panels A and B, **Table 1**). Prevalence increased progressively with age, from 4.23% (95% CI = 3.58–5.00) among individuals aged 30–34 years to 18.37% (95% CI = 15.90–21.13) among those aged 85–89 years. The prevalence was consistently higher in females than in males across all age groups. Among males, the overall prevalence of PAD was 4.76% (95% CI = 4.03–5.61), ranging from 3.73% (95% CI = 3.15–4.41) in those aged 30–34 years to 16.13% (95% CI = 13.91–18.64) in the 85–89 group. For females, the overall prevalence was 6.19% (95% CI = 5.25–7.27), ranging from 4.76% (95% CI = 4.02–5.62) to 19.91% (95% CI = 17.27–22.84), respectively. Of the 49.90 million estimated PAD cases, 43.73% were male (21.82 million; 95% CI = 18.48–25.73) and 56.27% were female (28.08 million; 95% CI = 23.85–33.00). We observed a distinct age-specific distribution, with the largest estimated case count in the 50–54 age group, totalling 5.98 million cases (95% CI = 5.06–7.05). The random-effects meta-analysis calculated the pooled prevalence of PAD to be 5.70% (95% CI = 4.93–6.52) (Figure S3 in the **Online Supplementary Document**), consistent with estimates derived from the modelling approach. The sensitivity analysis showed that the pooled prevalence of PAD varied from 5.46% (95% CI = 4.73–6.24) to 5.80% (95% CI = 5.01–6.63) after removing a single study at a time (Figures S4 in the **Online Supplementary Document**), but no single study had an excessive influence on the pooled prevalence. Visual inspection of funnel plot revealed asymmetry, indicated by significant Egger’s test result (*t* = 4.11, *P* < 0.001) (Figures S5 in the **Online Supplementary Document**).

**Table 1:** Estimated prevalence and number of cases of PAD among Chinese people aged 30–89 years in 2023, by age group and sex

	Prevalence of PAD, % (95%CI)			People living with PAD, million (95%CI)		
**Age group in years**	**Male**	**Female**	**Overall**	**Male**	**Female**	**Overall**
30–34	3.73 (3.15–4.41)	4.76 (4.02–5.62)	4.23 (3.58–5.00)	2.38 (2.01–2.81)	2.87 (2.43–3.39)	5.24 (4.43–6.20)
35–39	3.92 (3.31–4.63)	4.99 (4.22–5.89)	4.44 (3.75–5.24)	2.07 (1.75–2.45)	2.49 (2.11–2.94)	4.57 (3.86–5.39)
40–44	4.11 (3.47–4.85)	5.23 (4.43–6.17)	4.66 (3.94–5.50)	2.04 (1.73–2.42)	2.48 (2.10–2.92)	4.52 (3.83–5.34)
45–49	4.30 (3.63–5.08)	5.47 (4.64–6.45)	4.87 (4.12–5.75)	2.51 (2.12–2.97)	3.08 (2.61–3.63)	5.59 (4.73–6.60)
50–54	4.46 (3.77–5.27)	5.68 (4.81–6.69)	5.07 (4.29–5.98)	2.65 (2.24–3.14)	3.32 (2.81–3.91)	5.98 (5.06–7.05)
55–59	4.58 (3.88–5.41)	5.83 (4.94–6.87)	5.21 (4.41–6.14)	2.29 (1.94–2.70)	2.90 (2.46–3.42)	5.19 (4.40–6.12)
60–64	4.71 (3.99–5.56)	6.00 (5.08–7.06)	5.35 (4.53–6.31)	1.73 (1.46–2.04)	2.18 (1.85–2.56)	3.91 (3.31–4.61)
65–69	5.06 (4.28–5.97)	6.43 (5.45–7.57)	5.76 (4.88–6.78)	1.80 (1.53–2.13)	2.37 (2.01–2.79)	4.18 (3.54–4.92)
70–74	6.03 (5.11–7.10)	7.65 (6.50–8.98)	6.86 (5.83–8.07)	1.44 (1.22–1.70)	1.91 (1.63–2.25)	3.36 (2.85–3.94)
75–79	8.07 (6.86–9.46)	10.18 (8.69–11.89)	9.18 (7.83–10.74)	1.18 (1.00–1.38)	1.65 (1.41–1.93)	2.83 (2.41–3.32)
80–84	11.41 (9.76–13.30)	14.29 (12.28–16.57)	13.00 (11.15–15.10)	1.02 (0.87–1.19)	1.57 (1.35–1.82)	2.59 (2.22–3.01)
85–89	16.13 (13.91–18.64)	19.91 (17.27–22.84)	18.37 (15.90–21.13)	0.69 (0.60–0.80)	1.24 (1.08–1.43)	1.94 (1.68–2.23)
Overall (30–89)	4.76 (4.03–5.61)	6.19 (5.25–7.27)	5.47 (4.64–6.44)	21.82 (18.48–25.73)	28.08 (23.85–33.00)	49.90 (42.32–58.73)

CI – confidence interval, PAD – peripheral artery disease

**Figure 3:**
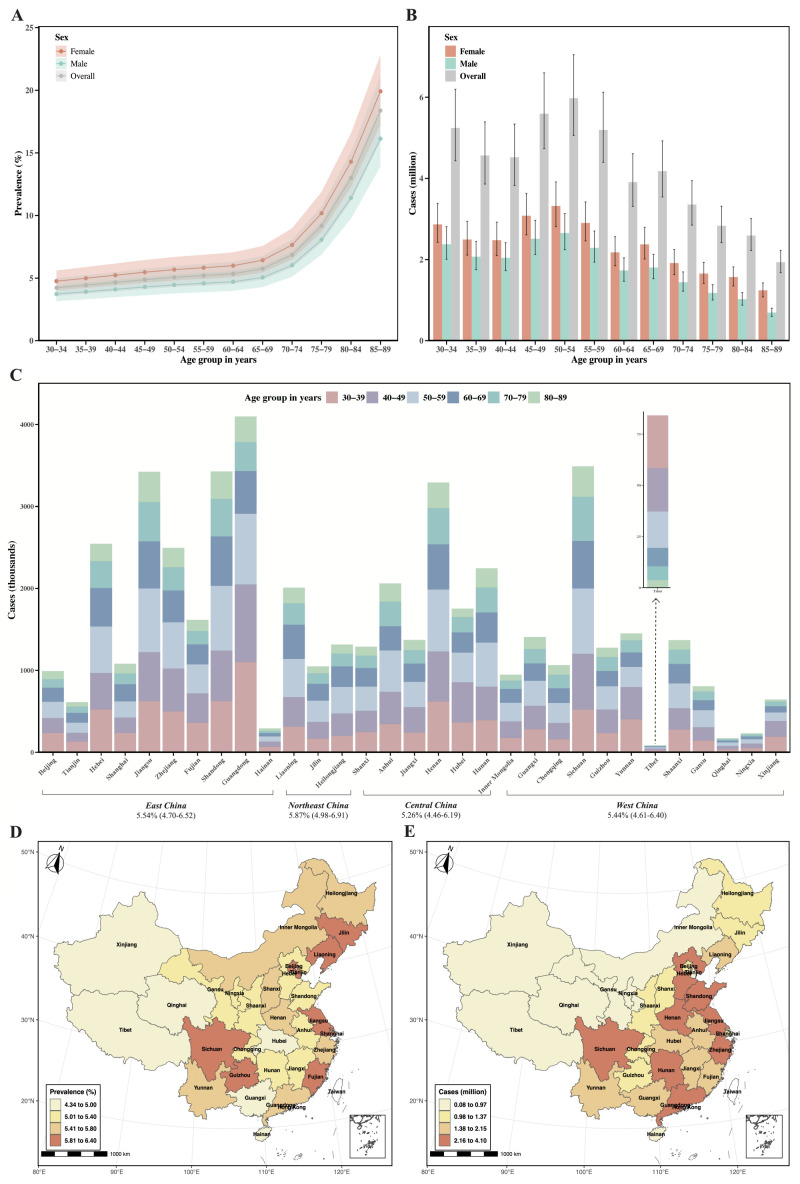
National, regional and provincial prevalence of PAD in 2023. **Panel A.** National age- and sex-specific prevalence. **Panel B.** National age- and sex-specific cases. **Panel C.** Regional prevalence and provincial cases and its contributing age groups in 2023. **Panel D.** Provincial prevalence. **Panel E.** Provincial cases. CI – confidence interval, PAD – peripheral artery disease.

Female sex was identified as a significant factor associated with PAD (OR = 1.53; 95% CI = 1.12–2.10), consistent with our sex-specific prevalence estimates for PAD in China. Current smoking (OR = 2.10; 95% CI = 1.25–3.55), hypertension (OR = 1.73; 95% CI = 1.37–2.18), diabetes (OR = 1.50; 95% CI = 1.22–1.84), coronary heart disease (OR = 1.50; 95% CI = 1.25–1.82) and stroke (OR = 1.86; 95% CI = 1.32–2.61) were also significantly associated with PAD (**Figure 3**; Table S9 in **Online Supplementary Document**).

We observed substantial geographic variation in PAD prevalence and cases across China’s four economic regions and 31 provinces (**Figure 2**, Panels C–E). Our model estimated Northeast China had the highest regional prevalence of PAD in 2023 (5.87%; 95% CI = 4.98–6.91), while Central China had the lowest (5.26%; 95% CI = 4.46–6.19). East China accounted for the largest share of PAD cases (20.58 million; 95% CI = 17.45–24.22), while Northeast China had the smallest (4.37 million; 95% CI = 3.71–5.15) (Table S10 in the **Online Supplementary Document**). At the provincial level, PAD prevalence was the highest in Beijing (6.40%; 95% CI = 5.43–7.53) and the lowest in Tibet (4.35%; 95% CI = 3.68–5.12). Guangdong recorded the largest number of national PAD cases (4.10 million; 95% CI = 3.47–4.83), while Tibet had the fewest (0.08 million; 95% CI = 0.07–0.10) (Table S11 in the **Online Supplementary Document**).

## DISCUSSION

This study provides the most comprehensive estimates of the prevalence of PAD in mainland China at the national, regional, and provincial levels to date. In 2023, the overall prevalence of PAD was 5.47%, affecting approximately 49.90 million Chinese adults aged 30–89 years. The burden of PAD increased progressively with age and showed notable disparities by sex and geographic region. Regionally, Northeast China had the highest regional prevalence (5.87%), while Central China had the lowest (5.26%); provincially, Beijing had the highest prevalence (6.40%), while Tibet had the lowest (4.35%).

The prevalence of PAD among individuals aged 30–89 years in mainland China in 2023 was estimated at 5.47%, corresponding to 49.90 million cases. This substantial burden may reflect several factors, including population ageing, urbanisation, the growing prevalence of noncommunicable diseases such as diabetes and hypertension, and changes in modelling strategies [8,9]. Although the estimated overall prevalence of PAD may appear relatively low, the large absolute number of affected individuals highlight the magnitude of this public health issue in China. Together, these findings highlight PAD as an increasingly important public health challenge and reinforce the need for earlier detection and intervention.

This burden was also unevenly distributed by sex. Specifically, while we observed an upward trend among both sexes (ranging from 4.23% to 18.37%), PAD was more prevalent in females than in males (6.19% *vs*. 4.76%). This finding was further supported by the meta-analysis of associated factors, which identified female sex as a marked risk factor for PAD, with a meta-OR of 1.53. One potential explanation of this disparity relates to sex hormones, particularly oestrogen, which exerts vasculoprotective effects through enhancement of endothelial function and attenuation of inflammation [20]. Following menopause, oestrogen loss may accelerate atherosclerosis and increase the risk of PAD in women [21–25]. Furthermore, sex-based differences in vascular structure, including smaller vessel diameter and less developed collateral circulation, may worsen ischaemic injury and hinder recovery [26]. Interestingly, despite males having a higher prevalence of major PAD risk factors (*e.g.* smoking, hypertension, diabetes), PAD is more common in females [27]. This apparent paradox may reflect sex-specific differences in susceptibility to these risk factors. For example, diabetes and hyperlipidaemia have been reported to increase the risk of intermittent claudication four-fold in women [28,29]. The clinical presentation of PAD also differs by sex: males with PAD are more likely to present with claudication symptoms, while females are more likely to have asymptomatic PAD or advanced critical limb ischaemia, conditions associated with potentially greater harm [30]. Greater awareness among clinicians and the public is needed, together with further research that would clarify sex-specific risk factors, disease burden, and outcomes. Strengthening early detection and timely intervention will be essential to reducing sex-based disparities in outcomes.

The analysis of associated factors identified female sex, current smoking, hypertension, and diabetes as being positively associated with the risk of PAD, consistent with findings from the China PAD Study (2019) [14]. We further examined five additional associated factors and identified two significant contributors: coronary heart disease and stroke. However, to maintain consistency with the previous study [14], and considering the availability of provincial prevalence data, clinical significance, and evidence strength, we only incorporated current smoking, hypertension, and diabetes into the associated factor-based model to estimate provincial case numbers. These factors are widely recognised by clinical guidelines as critical targets for PAD management [31]. Among these, tobacco exposure, particularly cigarette smoking, remains the strongest risk factor for PAD progression and complications [32]. Evidence supports smoking cessation interventions to improve vascular health [33]. Diabetes is another key factor, with individuals having both diabetes and PAD facing a three- to four-fold increase in mortality and a five-fold higher risk of amputation compared to PAD alone [34]. Similarly, managing hypertension is essential for reducing cardiovascular risk in PAD patients [35]. Despite these findings, inconsistent definitions across studies and limited data points for meta-analyses of other potential associated factors underscore the need for the re-evaluation of these variables in future studies to strengthen the evidence base. These findings emphasise the importance of addressing modifiable risk factors through targeted interventions and validate the scientific rigor and practical utility of the associated factor-based model, further highlighting its relevance for public health strategies.

Lastly, we observed substantial geographical variations. Among the four economic regions, Northeast China had the highest regional prevalence, while Central China had the lowest. Provincially, Beijing reported the highest prevalence, while Tibet had the lowest. These disparities are largely driven by differences in demographic structures and exposure to key associated factors. For instance, provinces in Northeast China exhibit relatively high rates of current smoking, hypertension, and diabetes [36–38]. The prevalence of hypertension and diabetes in Beijing, meanwhile, far exceeded the national average [36,38]. In contrast, Tibet had the lowest prevalence of diabetes among all 31 provinces in mainland China [36]. Despite the high prevalence observed in Northeast China and Beijing, the absolute number of PAD cases was concentrated in East China and populous provinces like Guangdong. Understanding the epidemiology of PAD at regional and provincial levels, therefore, remains essential to developing tailored strategies and informing guidelines for PAD prevention and control

The key strengths of this study include a comprehensive search strategy, dual-review process, stringent selection criteria, and the integration of multiple data sources, resulting in the inclusion of 54 unique articles. The application of an age-sex splitting procedure further enriched the dataset, enabling more granular analyses and reliable extrapolations across all regions and provinces. The scientific rationale for the modelling approach used here lies in its foundation at the national level, where age- and sex-specific prevalence data from each included article were synthesised to construct a unified prevalence pattern. Given the well-established association of PAD with age and sex, as supported by this study and prior evidence [7,14], the resulting curve represents the natural progression of PAD and is adaptable to specific locations. Additionally, our analysis incorporated data on associated factors, examined using multivariable logistic regressions to minimise the potential bias inherent in univariable analyses. These factors provided valuable insights into the drivers of PAD prevalence and facilitated a detailed evaluation at the provincial level. Finally, building on our earlier work using the GHERG approach, we generated updated 2023 estimates informed by newly available evidence and improved modelling. The adoption of standardised PAD definitions reduced uncertainty arising from inconsistent case definitions, thereby strengthening the robustness of the prevalence estimates.

Several limitations should be acknowledged. First, despite efforts to standardise diagnostic criteria and apply consistent definitions across included articles, heterogeneity in study design, measurement protocols, population characteristics, and settings may have introduced unmeasured bias. Second, regional coverage was uneven; most studies were conducted in East and West China, while Northeast China was underrepresented, while certain provinces in West and Central China (*e.g.* Tibet and Henan) contributed no data. Priority should be given to expanding epidemiological data collection for PAD in underserved regions by adopting standardised diagnostic criteria. Third, our age-sex-splitting procedure relied on a derived national age- and sex-specific prevalence pattern, which introduced partial model dependency. Although the procedure incorporated study-specific demographic information, its accuracy was limited by the uneven distribution and incomplete stratification of the available primary data. Additionally, the estimation of regional and provincial prevalence relied on an associated factor-based model that incorporated demographic variables (age, sex, study year) and key associated factors (current smoking, hypertension, diabetes), but may have overlooked the contribution of other relevant factors. Since the included articles were unevenly distributed across China, there were insufficient data to derive robust province-specific or regional ORs. We, therefore, used nationally pooled ORs in the model, and these provincial estimates should be interpreted as model-based approximations rather than direct observations. Differences between provinces should also be interpreted cautiously. Future studies using more geographically representative ABI-based data are needed to validate and refine these subnational estimates and to assess how temporal changes in major PAD associated factors may have contributed to the burden of PAD in China.

Our findings provide valuable insights into the epidemiology of PAD and carry important policy implications for China. Importantly, this work is more than a simple update of previous reviews. Although earlier studies provided national estimates and limited subnational data, the burden of PAD in China has remained insufficiently characterised at the provincial level. This gap is especially relevant in a country with substantial geographic, demographic, and epidemiological heterogeneity, where national averages may mask marked local variation. Using a systematic review and an established modelling framework developed by GHERG, we generated up-to-date estimates of PAD prevalence at the national, regional, and, for the first time, provincial levels, thereby addressing an important gap in subnational evidence. The contribution of this study lies primarily in the systematic synthesis of the available evidence and its use to produce comparable estimates across geographic levels. In the absence of directly comparable provincial survey data, these model-based estimates provide a practical basis for public health decision-making. Furthermore, by generating evidence-based prevalence estimates for regions lacking original survey data, this modelling approach may be informative for other countries with sparse surveillance data. Within China, these estimates may also help inform the allocation of local health resources.

## CONCLUSIONS

Our study provides a comprehensive assessment of the prevalence of PAD in mainland China, revealing pronounced age-related trends, sex differences, and regional disparities, which underscore the substantial public health burden of the disease. We observed that PAD was more prevalent among elderly individuals, females, current smokers, and those with hypertension or diabetes in the Chinese population. The highest prevalence was observed in Northeast China and the lowest in Central China, highlighting the need for region-specific interventions. Targeted efforts to address modifiable risk factors, such as smoking and hypertension, alongside strategies to enhance early detection and management, are essential to reduce the long-term adverse outcomes of PAD.

## Additional material


Online Supplementary Document
