# Supplementary material for: National, regional and provincial prevalence of peripheral artery disease in Chinese adults in 2023: an updated systematic review and modelling study
Source: J Glob Health. 2026 Apr 17;16:04155. doi: 10.7189/jogh.16.04155 (PMC13086483; doi:10.7189/jogh.16.04155)
Supplement: Online Supplementary Document [file jogh-16-04155-s001.pdf]

Supplement to: Zhou J, Shan S, Shen G, Tan B, Ying J, Rudan I, Song P; Global Health Epidemiology Research Group (GHERG). National, regional and provincial prevalence of peripheral artery disease in Chinese adults in 2023: an updated systematic review and modelling study. J Glob Health. 2026;16:04155.

Section 1. PRISMA checklist 2
Section 2. GATHER reporting checklist 5
Section 3. Search strategy to identify studies reporting the prevalence of peripheral artery disease 6
Section 4. eMethods: The detailed description of stages used to derive the national, regional and provincial prevalence of peripheral artery disease 8
Stage 1: Data preparation 8
Stage 1.1: Age- and sex-specific prevalence patterns 8
Stage 1.2: Age- and sex-splitting 9
Stage 2: National age- and sex- specific prevalence of peripheral artery disease 9
Stage 3: Regional and provincial age- and sex- specific prevalence of peripheral artery disease 10
Stage 3.1: Meta-analysis of factors associated with peripheral artery disease 10
Stage 3.2: Provincial age- and sex- specific prevalence of peripheral artery disease 10
Stage 3.3: Regional age- and sex- specific prevalence of peripheral artery disease 11
Section 5. Supplementary tables and figures 12
Table S1. The time-lag between investigation and publication in the included articles (N=54) 12
Table S2. Quality assessment scale by the Joanna Briggs Institute Critical Appraisal Checklist for Prevalence Studies 13
Table S3. Mixed-effects meta-regression models for rate patterns of the peripheral artery disease prevalence 15
Table S4. Multilevel mixed-effects meta-regression models for peripheral artery disease prevalence in China 16
Table S5. Criteria for quality of evidence classification in observational studies 17
Table S6. Four economic regions in the mainland of China 18
Table S7. Summary of the main characteristics of the included studies (N=54) 19
Table S8. Detailed characteristics of the included articles (N=54) 20
Table S9. Included studies in meta-analysis of associated factors of peripheral artery disease in China 23
Table S10. Estimated age- and sex-specific prevalence and number of cases of peripheral artery disease by economic regions in the mainland of China in 2023 26
Table S11. Estimated provincial prevalence and number of cases of peripheral artery disease in the mainland of China in 2023 29
Figure S1. Rate pattern for peripheral artery disease prevalence in China 31
Figure S2. Quality scores of the included articles using the Joanna Briggs Institute Critical Appraisal Checklist for Prevalence Studies 32
Figure S3. Pooled prevalence of peripheral artery disease in China (N=54) 35
Figure S4. Leave-one-out sensitivity analysis of peripheral artery disease prevalence in China (N=54) 36
Figure S5. Publication bias in studies on peripheral artery disease prevalence in China (N=54) 37
Section 6. Articles retained for analysis (N=54) 38

Section 1. PRISMA checklist

Section and Topic	Item #	Checklist item	Location where item is reported
**TITLE**			
Title	1	Identify the report as a systematic review.	1
**ABSTRACT**			
Abstract	2	See the PRISMA 2020 for Abstracts checklist.	2
**INTRODUCTION**			
Rationale	3	Describe the rationale for the review in the context of existing knowledge.	3
Objectives	4	Provide an explicit statement of the objective(s) or question(s) the review addresses.	3
**METHODS**			
Eligibility criteria	5	Specify the inclusion and exclusion criteria for the review and how studies were grouped for the syntheses.	4
Information sources	6	Specify all databases, registers, websites, organisations, reference lists and other sources searched or consulted to identify studies. Specify the date when each source was last searched or consulted.	4
Search strategy	7	Present the full search strategies for all databases, registers and websites, including any filters and limits used.	4
Selection process	8	Specify the methods used to decide whether a study met the inclusion criteria of the review, including how many reviewers screened each record and each report retrieved, whether they worked independently, and if applicable, details of automation tools used in the process.	4
Data collection process	9	Specify the methods used to collect data from reports, including how many reviewers collected data from each report, whether they worked independently, any processes for obtaining or confirming data from study investigators, and if applicable, details of automation tools used in the process.	4
Data items	10a	List and define all outcomes for which data were sought. Specify whether all results that were compatible with each outcome domain in each study were sought (e.g. for all measures, time points, analyses), and if not, the methods used to decide which results to collect.	4
	10b	List and define all other variables for which data were sought (e.g. participant and intervention characteristics, funding sources). Describe any assumptions made about any missing or unclear information.	4
Study risk of bias assessment	11	Specify the methods used to assess risk of bias in the included studies, including details of the tool(s) used, how many reviewers assessed each study and whether they worked independently, and if applicable, details of automation tools used in the process.	4-5

Section and Topic	Item #	Checklist item	Location where item is reported
Effect measures	12	Specify for each outcome the effect measure(s) (e.g. risk ratio, mean difference) used in the synthesis or presentation of results.	5-7
Synthesis methods	13a	Describe the processes used to decide which studies were eligible for each synthesis (e.g. tabulating the study intervention characteristics and comparing against the planned groups for each synthesis (item #5)).	5-7
	13b	Describe any methods required to prepare the data for presentation or synthesis, such as handling of missing summary statistics, or data conversions.	5-7
	13c	Describe any methods used to tabulate or visually display results of individual studies and syntheses.	5-7
	13d	Describe any methods used to synthesize results and provide a rationale for the choice(s). If meta-analysis was performed, describe the model(s), method(s) to identify the presence and extent of statistical heterogeneity, and software package(s) used.	5-7
	13e	Describe any methods used to explore possible causes of heterogeneity among study results (e.g. subgroup analysis, meta-regression).	5-7
	13f	Describe any sensitivity analyses conducted to assess robustness of the synthesized results.	5-7
Reporting bias assessment	14	Describe any methods used to assess risk of bias due to missing results in a synthesis (arising from reporting biases).	4-5
Certainty assessment	15	Describe any methods used to assess certainty (or confidence) in the body of evidence for an outcome.	4-5
**RESULTS**			
Study selection	16a	Describe the results of the search and selection process, from the number of records identified in the search to the number of studies included in the review, ideally using a flow diagram.	7, Figure2
	16b	Cite studies that might appear to meet the inclusion criteria, but which were excluded, and explain why they were excluded.	7, Figure2, Appendix
Study characteristics	17	Cite each included study and present its characteristics.	7, Appendix
Risk of bias in studies	18	Present assessments of risk of bias for each included study.	7, Appendix
Results of individual studies	19	For all outcomes, present, for each study: (a) summary statistics for each group (where appropriate) and (b) an effect estimate and its precision (e.g. confidence/credible interval), ideally using structured tables or plots.	7-8, Table1, Appendix
Results of syntheses	20a	For each synthesis, briefly summarise the characteristics and risk of bias among contributing studies.	7-8, Appendix
	20b	Present results of all statistical syntheses conducted. If meta-analysis was done, present for each the summary estimate and its precision (e.g. confidence/credible interval) and measures of statistical heterogeneity. If comparing groups, describe the direction of the effect.	7-8, Figure3-4, Table1, Appendix

Section and Topic	Item #	Checklist item	Location where item is reported
	20c	Present results of all investigations of possible causes of heterogeneity among study results.	7-8, Appendix
	20d	Present results of all sensitivity analyses conducted to assess the robustness of the synthesized results.	8, Appendix
Reporting biases	21	Present assessments of risk of bias due to missing results (arising from reporting biases) for each synthesis assessed.	7, Appendix
Certainty of evidence	22	Present assessments of certainty (or confidence) in the body of evidence for each outcome assessed.	Figure3, Appendix
**DISCUSSION**			
Discussion	23a	Provide a general interpretation of the results in the context of other evidence.	8
	23b	Discuss any limitations of the evidence included in the review.	9-10
	23c	Discuss any limitations of the review processes used.	10
	23d	Discuss implications of the results for practice, policy, and future research.	10
**OTHER INFORMATION**			
Registration and protocol	24a	Provide registration information for the review, including register name and registration number, or state that the review was not registered.	4
	24b	Indicate where the review protocol can be accessed, or state that a protocol was not prepared.	4
	24c	Describe and explain any amendments to information provided at registration or in the protocol.	N/A
Support	25	Describe sources of financial or non-financial support for the review, and the role of the funders or sponsors in the review.	11
Competing interests	26	Declare any competing interests of review authors.	11
Availability of data, code and other materials	27	Report which of the following are publicly available and where they can be found: template data collection forms; data extracted from included studies; data used for all analyses; analytic code; any other materials used in the review.	11

From: Page MJ, McKenzie JE, Bossuyt PM, Boutron I, Hoffmann TC, Mulrow CD, et al. The PRISMA 2020 statement: an updated guideline for reporting systematic reviews. BMJ 2021;372:n71. doi: 10.1136/bmj.n71

Section 2. GATHER reporting checklist

Checklist of information that should be included in new reports of global health estimates

Item#	Checklist item	Reported <u>on page #</u>
**Objectives and funding**		
1	Define the indicator(s), populations (including age, sex, and geographic entities), and time period (s) for which estimates were made.	2, 3
2	List the funding sources for the work.	2, 11
**Data Inputs**		
For all data inputs from multiple sources that are synthesized as part of the study:		
3	Describe how the data were identified and how the data were accessed.	4, Appendix
4	Specify the inclusion and exclusion criteria. Identify all ad-hoc exclusions.	4, Appendix
5	Provide information on all included data sources and their main characteristics. For each data source used, report reference information or contact name/institution, population represented, data collection method, year(s) of data collection, sex and age range, diagnostic criteria or measurement method, and sample size, as relevant.	4-5, Appendix
6	Identify and describe any categories of input data that have potentially important biases (e.g., based on characteristics listed in item 5).	4-5, Appendix
For data inputs that contribute to the analysis but were not synthesized as part of the study:		
7	Describe and give sources for any other data inputs.	Appendix
For all data inputs:		
8	Provide all data inputs in a file format from which data can be efficiently extracted (e.g., a spreadsheet rather than a PDF), including all relevant meta-data listed in item 5. For any data inputs that cannot be shared because of ethical or legal reasons, such as third-party ownership, provide a contact name or the name of the institution that retains the right to the data.	N/A
**Data analysis**		
9	Provide a conceptual overview of the data analysis method. A diagram may be helpful.	Figure1, 5
10	Provide a detailed description of all steps of the analysis, including mathematical formulae. This description should cover, as relevant, data cleaning, data pre-processing, data adjustments and weighting of data sources, and mathematical or statistical model(s).	5-7, Appendix
11	Describe how candidate models were evaluated and how the final model(s) were selected.	5-7, Appendix
12	Provide the results of an evaluation of model performance, if done, as well as the results of any relevant sensitivity analysis.	5-7, Appendix
13	Describe methods for calculating uncertainty of the estimates. State which sources of uncertainty were, and were not, accounted for in the uncertainty analysis.	5-7, Appendix
14	State how analytic or statistical source code used to generate estimates can be accessed.	11, Appendix
**Results and Discussion**		
15	Provide published estimates in a file format from which data can be efficiently extracted.	7-8, Appendix
16	Report a quantitative measure of the uncertainty of the estimates (e.g. uncertainty intervals).	7-9
17	Interpret results in light of existing evidence. If updating a previous set of estimates, describe the reasons for changes in estimates.	8-9
18	Discuss limitations of the estimates. Include a discussion of any modelling assumptions or data limitations that affect interpretation of the estimates.	10

This checklist should be used in conjunction with the GATHER statement and Explanation and Elaboration document, found on gather-statement.org

Section 3. Search strategy to identify studies reporting the prevalence of peripheral artery disease

Database	Access date	Subject category	Sub-database	Search terms	Publication date	Search method
**CNKI**	12/8/2024	Medicine & Public Health	Journal, Featured journal, Doctoral dissertation, Master dissertation, Domestic conferences, International conferences	(SU%'外周动脉疾病'+'外周动脉病'+'外周动脉病变'+'外周动脉硬化'+'下肢动脉疾病'+'下肢动脉病'+'下肢动脉病变'+'下肢动脉硬化'+'周围动脉疾病'+'周围动脉硬化'+'peripheral artery disease') AND (SU%'发病率'+'发生率'+'患病率'+'罹患率'+'现患率'+'死亡率'+'病死率'+'流行'+'负担'+'现况调查'+'现况研究')	4/3/2017-12/8/2024	Comprehensive search: subject, title, keywords and abstract
**Wanfang**	12/8/2024	Not applicable	Journal article	(主题:(外周动脉疾病) OR 主题:(外周动脉病) OR 主题:(外周动脉病变) OR 主题:(外周动脉硬化) OR 主题:(下肢动脉疾病) OR 主题:(下肢动脉病) OR 主题:(下肢动脉病变) OR 主题:(下肢动脉硬化) OR 主题:(周围动脉疾病) OR 主题:(周围动脉硬化) OR 主题:(peripheral artery disease)) AND (主题:(发病率) OR 主题:(发生率) OR 主题:(患病率) OR 主题:(罹患率) OR 主题:(现患率) OR 主题:(死亡率) OR 主题:(病死率) OR 主题:(流行) OR 主题:(负担)OR 主题:(现况调查) OR 主题:(现况研究))	4/3/2017-12/8/2024	Comprehensive search: subject (including title, keywords and abstract)
**VIP**	12/8/2024	Medicine & Public Health	All journals	(M=(外周动脉疾病 OR 外周动脉病 OR 外周动脉病变 OR 外周动脉硬化 OR 下肢动脉疾病 OR 下肢动脉病 OR 下肢动脉病变 OR 下肢动脉硬化 OR 周围动脉疾病 OR 周围动脉硬化 OR peripheral artery disease)) AND (M=(发病率 OR 发生率 OR 患病率 OR 罹患率 OR 现患率 OR 死亡率 OR 病死率 OR 流行 OR 负担 OR 现况研究 OR 现况调查))	2017-2024	Comprehensive search: subject, title, keywords and abstract
**PubMed**	12/8/2024	Not applicable	Not applicable	((peripheral artery disease) AND (China OR Chinese) AND (inciden* OR prevalen* OR morbidity OR mortality OR epidemiology)) AND ("2017/03/04"[Date - Publication]: "2024/08/12"[Date - Publication])	4/3/2017-12/8/2024	Comprehensive search: all fields
**Embase**	12/8/2024	Not applicable	Not applicable	#1 'peripheral arterial disease'/exp or 'peripheral arterial disease' or 'peripheral artery disease'<br>#2 'china'/exp or 'chinese'/exp<br>#3 'incidence'/exp or 'inciden*'<br>#4 ‘prevalence’/exp or ‘prevalen*’	01/01/2017-12/8/2024	Comprehensive search: all fields

Database	Access date	Subject category	Sub-database	Search terms		Publication date	Search method
				#5	'morbidity'/exp or 'morbidity'		
				#6	'mortality'/exp or 'mortality'		
				#7	'epidemiology'/exp or 'epidemiolog*'		
				#8	#3 or #4 or #5 or #6 or #7		
				#9	#1 and #2 and #8		
				#10	#9 AND [embase]/lim NOT ([embase]/lim AND [medline]/lim) AND [2017-2024]/py		
**MEDLINE**	12/8/2024	Not applicable	Not applicable	#1	peripheral artery disease.mp. or exp Peripheral Arterial Disease/	01/01/2017-12/8/2024	Comprehensive search: all fields
				#2	Chin*.mp.		
				#3	incidence/ or inciden*.mp.		
				#4	prevalence/ or prevalen*.mp.		
				#5	morbidity/		
				#6	mortality/		
				#7	epidemiology/		
				#8	3 or 4 or 5 or 6 or 7		
				#9	1 and 2 and 8		
				#10	limit 9 to yr="2017 -Current"		

Number of records returned: 4222

Access date: 12 August 2024

Section 4. eMethods: The detailed description of stages used to derive the national, regional and provincial prevalence of peripheral artery disease

This section is a supplement to the Methods part in the main text.

Stage 1: Data preparation

Stage 1.1: Age- and sex-specific prevalence patterns

Using our systematic review and data extraction approach, multiple data points (e.g., age-specific or sex-specific prevalence) were extracted from the included articles. To account for the hierarchical structure of the data and the inclusion of multiple articles from the same province, we adopted a multilevel multivariable mixed-effects meta-regression approach and took study identification as the random-effects. Zero cells were replaced with a value of 0.0005 to allow for the inclusion of reported zero cases.

Given that:

$$prevalence = p = \frac{number\ of\ cases}{number\ of\ participants}$$

Then, the prevalence was stabilized with the logit link,

$$\text{logit}(p) = \ln(\frac{p}{1-p}) = \ln(odds) = \alpha + \beta_1 * x_1 + \beta_2 * x_2 + \cdots \beta_n * x_n + u_i$$

Thus,

$$odds = \frac{p}{1-p} = e^{(\alpha+\beta_1*x_1+\beta_2*x_2+\cdots\beta_n*x_n+u_i)}$$

And,

$$prevalence = p = \frac{e^{(\alpha+\beta_1*x_1+\beta_2*x_2+\cdots\beta_n*x_n+u_i)}}{1+e^{(\alpha+\beta_1*x_1+\beta_2*x_2+\cdots\beta_n*x_n+u_i)}}$$

where α is the intercept term, β is the coefficient, and x is the variable.

First, we assessed the effects of average age and female proportion via the univariable meta-regression, both were significantly associated with the peripheral artery disease (PAD) prevalence. Subsequently, the effects of other cluster-level variables $x_1 - x_n$, namely economic region, setting, and study year were assessed, but neither demonstrated a significant association (**Table S3 in the Section 5, p 15**). Bases on expertise and on the abovementioned explorations, we fitted a multilevel mixed-effects meta-regression to derive the "prevalence patterns" for PAD, with average age, and female proportion as fixed-effect variables and with study identification as the random-effect (u_i). Restricted cubic regression splines were performed to model the functional forms of the non-linear association of average age and PAD prevalence, with knots being selected by visual inspection at the inflection points of the curve (knots: 39.5, 57, 69.5, and 84.5). With the most data points were concentrated, we restricted estimates to 30-89 years.

Therefore,

$$\text{logit}(p) = \alpha + \beta_1 * Average\ age_1 + \beta_2 * Average\ age_2 + \beta_3 * Average\ age_3 + \beta_4 * Female\ proportion + u_i$$

Then,

$$\text{prevalence} = p = \frac{e^{(\alpha+\beta_1*Average\ age_1+\beta_2*Average\ age_2+\beta_3*Average\ age_3+\beta_4*Female\ proportion+u_i)}}{1+e^{(\alpha+\beta_1*Average\ age_1+\beta_2*Average\ age_2+\beta_3*Average\ age_3+\beta_4*Female\ proportion+u_i)}}$$

Where α is the intercept term, β is the coefficient, x is the variable, $Average\ age_1$-$Average\ age_3$ are variables generated in the process of fitting cubic splines, u_i represents the variance of random-effects.

Based on above steps, the "prevalence patterns" for PAD were generated (**Figure S1** in the **Section 5, p 31**).

Stage 1.2: Age- and sex-splitting

To improve data availability and comparability for prevalence modelling, an age-sex-splitting procedure was applied to transform aggregated prevalence data into age- and sex-specific estimates, based on the established "prevalence patterns" for PAD.

First, data labeled as "both" sex were split into male- and female-specific datapoints using the following equation:

$$C_{A,s} = (\sum_{a \in A} R_{a,s} N_{a,s}) \cdot P_{A,s}/N_{A,s} \cdot \frac{C_{A,S}}{\sum_{s \in S}((\sum_{a \in A} R_{a,s} N_{a,s}) \cdot P_{A,s}/N_{A,s})}$$

In this equation, s is the specific sex (male or female), S is the set of sexes that the data is aggregated across, a is a one-year age group, A is the set of ages the data is aggregated across, $C_{A,S}$ is the reported total case number to be split, $R_{a,s}$ is the prevalence in age group a and sex s from "prevalence patterns", $N_{a,s}$ is the population in age group a and sex s based on the 2020 population census of China, $P_{A,s}$ is the proportion of sex s in study sample, $N_{A,s}$ is the population in age group A and sex s based on the 2020 population census of China, and $C_{A,s}$ is the split case number of PAD in sex s.

Subsequently, datapoints with inconsistent age groups were standardized into uniform one-year age groups. Due to the absence of precise age-specific distribution data in the included studies, we applied an exponential adjustment method with numerical optimization to simulate age distributions that aligned with both the national age distribution and the sample's average age.

An adjustment parameter adj was introduced to modify the national age distribution through an exponential function:

$$Adjusted\ P_a = \frac{P_a \cdot e^{adj(a-\mu)}}{\sum_{a \in A} P_a \cdot e^{adj(a-\mu)}}$$

where a is a one-year age group, A is the set of ages the data is aggregated across, adj is the adjustment parameter, μ is the average age of reported sample, P_a is the proportion of age a in the 2020 population census of China, and $Adjusted\ P_a$ is the proportion of age a after adjustment. This adjustment increases the proportion of ages above μ if adj>0 and increases the proportion of ages below μ if adj<0.

The optimal value of adj were estimated using the Brent optimization method to minimize the squared difference between the average age of the adjusted distribution and the reported average age:

$$min_{adj} \left(\sum_{a \in A} Adjusted\ P_a \cdot a - \mu \right)^2$$

The optimization process was constrained to $adj \in [-10, 10]$ to ensure numerical stability.

After obtaining the optimal adj, we generated the adjusted age distribution $Adjusted\ P_a$. From this distribution, ages were sampled to create a simulated population that aligned with both the national age distribution and the target mean age.

Subsequently, a sex split was performed based on the adjusted age distribution:

$$C_a = R_a N_a \frac{C_A}{\sum_{a \in A} R_a N_a}$$

In this equation, a is a one-year age group, A is the set of ages the data is aggregated across, C_A is the reported total case number in ages A to be split, R_a is the prevalence in age group a from "prevalence patterns", N_a is the population in age group a based on the simulated population, and C_a is the split case number of PAD in age group a.

Following the age-sex splitting process, PAD case numbers from various articles were stratified into single-year age groups and disaggregated by sex (male and female).

Stage 2: National age- and sex- specific prevalence of peripheral artery disease

To systematically address heterogeneity across studies and account for hierarchical data structures,

multilevel mixed-effects meta-regression models were applied using age- and sex-specific PAD data. Data points falling outside the 95% prediction interval (PI) were identified as outliers and excluded from further analysis. We stabilized the prevalence by the logit link. Thus,

$$prevalence = p = \frac{e^{(\alpha+\beta_1*x_1+\beta_2*x_2+\cdots\beta_n*x_n+u_i)}}{1+e^{(\alpha+\beta_1*x_1+\beta_2*x_2+\cdots\beta_n*x_n+u_i)}}$$

where α is the intercept term, β is the coefficient, u_i represents the variance of random-effects, and x is the variable.

Given the purpose of the age-sex splitting procedure was to enrich the dataset and derive more robust and granular estimates, we followed the findings from variable exploration in ***Stage 1.1***. Consequently, to estimate the national prevalence of PAD among individuals aged 30-89 years, only average age and female proportion were included as fixed-effect variables in the final national model (**Table S4** in the **Section 5, p 16**), with knots positioned at 39.5, 57, 69.5, and 84.5.

Therefore,

$$\text{prevalence} = p = \frac{e^{(\alpha+\beta_1*Average\ age_1+\beta_2*Average\ age_2+\beta_3*Average\ age_3+\beta_4*Female\ proportion+u_i)}}{1+e^{(\alpha+\beta_1*Average\ age_1+\beta_2*Average\ age_2+\beta_3*Average\ age_3+\beta_4*Female\ proportion+u_i)}}$$

where α is the intercept term, β is the coefficient, and u_i represents the variance of random-effects.

Based on the above models, the national age-and sex-specific prevalence of PAD was generated (**Table S4** in the **Section 5, p 16**). To estimate the 2023 population for each province, the 2020 population census of China was used as a baseline, which ensures that the age and sex structure remained consistent with the 2020 census data. Assuming a uniform growth rate across all provinces, the 2023 provincial populations were calculated by multiplying each province's 2020 census population by the national growth rate, derived as the ratio of the 2023 UNPD population estimate to the 2020 census population. Using these population estimates, the national number of PAD cases ("national envelopes") among Chinese adults aged 30–89 years in 2023 was estimated by applying the age- and sex-specific prevalence rates.

Stage 3: Regional and provincial age- and sex- specific prevalence of peripheral artery disease

Stage 3.1: Meta-analysis of factors associated with peripheral artery disease

A subset of included articles further investigated factors associated with PAD using multivariable analysis, and only factors with similar definitions were considered. A random-effects (DerSimonian and Laird) meta-analysis was performed to synthesize the effects of associated factors with at least three informative data points, including demographic, anthropometric, lifestyle, and clinical factors. As previously summarized, we applied an established framework for observational studies (detailed protocols are provided in **Table S5** in the **Section 5, p 17**) to grade the credibility of evidence for these factors. Based on this framework, the evidence was classified into five levels: I, convincing; II, highly suggestive; III, suggestive; IV, weak and V, nonsignificant.

Stage 3.2: Provincial age- and sex- specific prevalence of peripheral artery disease

The national number of PAD cases among Chinese adults aged 30-89 years in 2023 was distributed into 31 provinces in the mainland of China through an "associated factor-based model", which was initially proposed by Global Health Epidemiology Reference Group and has been widely applied to estimate disease burden at global, regional, and national levels. Based on data availability and statistical significance, three associated factors synthesized in ***Stage 3.1***, namely current smoking, hypertension, and diabetes, were selected and incorporated into the "associated factor-based model". The provincial PAD cases ("province envelopes") were then imputed using the following formula:

$$N_{province} = Pop_{province} \times Prev_{PAD_{nation}} \times \left\{1 + \sum_{RF_1}^{RF_3}\left[\left(Prev_{RF_{province}} - Prev_{RF_{nation}}\right) \times \left(OR_{RF} - 1\right)\right]\right\}$$

Where $N_{province}$ and $Pop_{province}$ represent the number of PAD cases and population size, respectively, among adults aged 30-89 years in each province or municipality. $Prev_{PAD_{nation}}$ denotes the estimated national prevalence of PAD calculated in ***Stage 2***, while RF_1-RF_3 refer to the three selected associated

factors: current smoking, hypertension, and diabetes. $Prev_{RF_{province}}$ and $Prev_{RF_{nation}}$ are the prevalence rates of the three associated factors in each province or municipality and at the national level. OR_{RF} is the synthesized OR of current smoking, hypertension, and diabetes from ***Stage 3.1***.

To ensure that the sum of provincial cases aligned with the "national envelopes", an "adjustment index" was applied for each province. The adjusted provincial prevalence of PAD was then calculated by dividing the number of PAD cases in each province or municipality by its corresponding population.

Stage 3.3: Regional age- and sex- specific prevalence of peripheral artery disease

Finally, we developed "regional envelopes" for PAD by aggregating the cases within each economic region (East China, Northeast China, Central China, and West China). The regional prevalence of PAD was calculated as the number of PAD cases in each economic region divided by its corresponding population.

Given that the prevalence of the three associated factors in the "associated factor-based model" was only available for the 31 provinces of the mainland of China, Taiwan (Province of China), Hong Kong, and Macau were included solely to provide age- and sex-specific prevalence trends but were excluded from the calculation of case numbers at the national, regional, or provincial levels.

All analyses were performed in R, version 4.4.2. A two-sided *p*-value of less than 0.05 was indicative of statistical significance.

Section 5. Supplementary tables and figures

Table S1. The time-lag between investigation and publication in the included articles (N=54)

Study ID	Author (s)	Year published	Year investigated	Time-lag (year)
**P-01**	Liu Chengguo, et al.	2005	2004	1
**P-02**	Chuang Shao-Yuan, et al.	2005	2002	3
**P-03**	Zheng Liqiang, et al.	2006	2005	1
**P-04**	He Yao, et al.	2006	2001	5
**P-05**	J. Woo, et al.	2006	2006	0
**P-06**	Liu Hao, et al.	2007	2005	2
**P-07**	Wang Linglin, et al.	2007	2006	1
**P-08**	Samuel Y.S. Wong, et al.	2007	2002	5
**P-09**	Wang Yong, et al.	2009	2008	1
**P-10**	Zhou Yueying, et al.	2009	2008	1
**P-11**	Samuel Y.S. Wong, et al.	2009	NA	NA
**P-12**	Chen Jinghua, et al.	2010	NA	NA
**P-13**	An Wei, et al.	2010	NA	NA
**P-14**	Zhang Xu, et al.	2010	2009	1
**P-15**	Yang Xiaomin, et al.	2010	2009	1
**P-16**	Wang Guipeng, et al.	2010	2008	2
**P-17**	Xu Songqing, et al.	2010	2008	2
**P-18**	Xie Xiaoliang, et al.	2010	2007	3
**P-19**	Xiang Yang, et al.	2011	2010	1
**P-20**	Zhou Lin, et al.	2012	2010	2
**P-21**	Bai Xueqin, et al.	2012	2008	4
**P-22**	J. Woo, et al.	2012	2001	11
**P-23**	Chen Ping.	2012	2009	3
**P-24**	Liu Lu.	2013	NA	NA
**P-25**	Zhu Hongmin.	2013	NA	NA
**P-26**	Wang Lanying, et al.	2013	2010	3
**P-27**	Hu Bang-Chuan, et al.	2013	2006	7
**P-28**	Liu Yuping, et al.	2014	2011	3
**P-29**	Liang Yajun, et al.	2014	2011	3
**P-30**	Yang Jinghui.	2014	2010	4
**P-31**	Lin Liming, et al.	2014	2009	5
**P-32**	Han Hongfeng, et al.	2014	2011	3
**P-33**	Pan Xinhua, et al.	2015	NA	NA
**P-34**	Lin Chih-Hsueh, et al.	2015	2009	6
**P-35**	Wen Jiangping, et al.	2015	2007	8
**P-36**	Wang Anxin, et al.	2016	2011	5
**P-37**	Huang Shan.	2016	NA	NA
**P-38**	Wang Po, et al.	2017	2009	8
**P-39**	Lu Liya, et al.	2017	2006	11
**P-40**	Mao Yong, et al.	2017	2008	9
**P-41**	Fan Xiaohong, et al.	2017	2008	9
**P-42**	Wang Zengwu, et al.	2018	2014	4
**P-43**	Pan Jing, et al.	2018	2006	12
**P-44**	Zhao Qiannan, et al.	2019	2012	7
**P-45**	Cao Jingjing.	2019	2013	6
**P-46**	Wang Dandan, et al.	2020	2018	2
**P-47**	Huang Shujing, et al.	2021	2018	3
**P-48**	Zheng Yue, et al.	2023	2020	3
**P-49**	Wang Yong, et al.	2023	2016	7
**P-50**	Wang Yaxing, et al.	2023	2011	12
**P-51**	Fu Xiya, et al.	2023	2020	3
**P-52**	Song Xiaohui, et al.	2023	2017	6
**P-53**	Guo Qianlan.	2023	2018	5
**P-54**	Shang Qinggang, et al.	2024	2021	3

Note: The average time-lag was 4.40 based on 47 articles with available data.

Table S2. Quality assessment scale by the Joanna Briggs Institute Critical Appraisal Checklist for Prevalence Studies

Criterion		Descriptions for each criterion
**Q1**	Was the sample frame appropriate to address the target population?	It should include a broadly representative sample of the general population to report on the prevalence of PAD. Studies limited to non-representative subgroups, such as patients with diabetes, individuals at high risk for cardiovascular diseases, or those with other specific conditions, are not appropriate. Additionally, studies addressing non-systemic vascular conditions, such as intracranial or pulmonary vascular diseases, should be excluded.
**Q2**	Were study participants sampled in an appropriate way?	Participants should be selected using random probabilistic sampling methods from a broadly representative general population. For studies employing cluster sampling, the sampling methods must be clearly described, and the clustering effect should be accounted for in prevalence estimates. Random sampling is not required if all individuals are included from a well-defined and representative sample frame (e.g., a census or a database encompassing the entire target population). However, studies using convenience sampling methods, such as street surveys, public gathering interviews, or other non-representative approaches, are inappropriate because they fail to provide a representative sample of the target population.
**Q3**	Was the sample size adequate?	Studies should ideally include a sample size calculation to determine the number of participants required for reliable estimates of PAD prevalence. Larger sample sizes are preferred. If the study is based on a large national survey or registry that sufficiently represents the target population, a formal sample size calculation may not be necessary. In cases where no sample size calculation is reported and the study is not based on a large national survey or registry, investigators may need to assess the adequacy of the sample size using standard formulas.
**Q4**	Were the study subjects and the setting described in detail?	The study should report key demographic characteristics of participants, including age distribution, sex, and relevant sociodemographic factors. The geographic location, environmental context, and timeframe should also be detailed. In addition, the study should specify how and where measurements were conducted.
**Q5**	Was the data analysis conducted with sufficient coverage of the identified sample?	Response rates should be assessed across subgroups to identify any disproportionately low rates that could introduce coverage bias. For subgroup analyses, the sample sizes within each subgroup must be adequate to ensure reliable prevalence estimates.
**Q6**	Were valid methods used for the identification of the condition?	Studies should apply widely accepted definitions and diagnostic criteria. PAD status was assessed using validated devices. Specifically, studies must define PAD as having an ABI of less than 0.90 or 0.90 or less, rather than relying on typical symptoms or self-reported diagnoses.
**Q7**	Was the condition measured in a standard, reliable way for all participants?	The study should ensure that ABI measurements were conducted using consistent protocols across the entire population. This includes the use of properly sized cuffs, appropriate rest periods, and standardized measurement conditions. All data collectors must undergo appropriate training to ensure strict adherence to standardized procedures. The ABI measurement process must be uniform for all participants.
**Q8**	Was there appropriate statistical analysis?	The numerator (e.g., number of individuals with PAD) and denominator (e.g., total sample size) should be explicitly reported for all prevalence calculations. Prevalence estimates and percentages must be accompanied by CIs to reflect the precision of the results. The methods section should clearly describe the statistical techniques employed, the measurement of variables, and how

Criterion		Descriptions for each criterion
		data issues were addressed. The selected statistical methods must align with the study objectives and the characteristics of the data. Confounding variables (e.g., age, sex, socioeconomic status) should be adjusted for using appropriate techniques. For subgroup analyses, the methods should be detailed, and subgroup sample sizes must be sufficient to ensure reliable results.
**Q9**	Was the response rate adequate, and if not, was the low response rate managed appropriately?	The study should clearly report the overall response rate, in this analysis, 80% were deemed adequate. Reasons for non-response (e.g., refusals, dropouts, or "not founds") should be analysed and discussed. For studies with low response rates, the validity of the findings should be justified by demonstrating that non-responders did not introduce significant bias. Strategies to address low response rates, such as statistical adjustments (e.g., weighting or imputation) or measures to increase participation (e.g., follow-ups or incentives), should be clearly described.

Note: Descriptions for each criterion were tailored specifically to this study. Each criterion was assessed as Yes (1), No (0), or Unclear (0). A total quality score ranging from zero to nine was assigned to each study, with larger scores indicating better methodological quality. PAD, peripheral artery disease; ABI, ankle-brachial index; CI, confidence interval.

Table S3. Mixed-effects meta-regression models for rate patterns of the peripheral artery disease prevalence

Variable	Number of data points	β (95% CI)	*P*-value
**Univariable meta-regression**			
Average age	202	0.0236 (0.0214, 0.0259)	**<0.0001**
Female proportion	202	0.2364 (0.1929, 0.2798)	**<0.0001**
**Age- and sex-adjusted meta-regression**			
Economic region			
East	124	Reference	
West	40	-0.2205 (-0.5784, 0.1374)	0.2272
Setting			
Mixed	59	Reference	
Rural	63	-0.0237 (-0.4587, 0.4113)	0.9151
Urban	80	0.0860 (-0.2883, 0.4603)	0.6524
Study year	202	0.0130 (-0.0207, 0.0466)	0.4500
**Final multilevel meta-regression**			
Intercept	202	-3.6570 (-3.9738, -3.3403)	**<0.0001**
Average age			
Age_1	202	0.0088 (0.0030, 0.0146)	**0.0028**
Age_2	202	0.0001 (-0.0184, 0.0185)	**0.9950**
Age_3	202	0.1552 (0.0759, 0.2344)	**0.0001**
Female proportion	202	0.2546 (0.2111, 0.2981)	**<0.0001**

Note: Age_1-Age_3 were variables generated in the process of restricted cubic spline, and the knots were 39.5, 57, 69.5, and 84.5; CI, confidence interval.

Table S4. Multilevel mixed-effects meta-regression models for peripheral artery disease prevalence in China

Variable	Number of data points	*β* (95% CI)	*P*-value
Intercept	4372	-3.5696 (-3.7446, -3.3946)	**<0.0001**
Average age			
Age_1	4372	0.0100 (0.0099, 0.0101)	**<0.0001**
Age_2	4372	-0.0125 (-0.0128, -0.0122)	**<0.0001**
Age_3	4372	0.1935 (0.1923, 0.1946)	**<0.0001**
Female proportion	4372	0.2541 (0.2536, 0.2545)	**<0.0001**

Note: Age_1-Age_3 were variables generated in the process of restricted cubic spline, and the knots were 39.5, 57, 69.5, and 84.5; CI, confidence interval.

.

Table S5. Criteria for quality of evidence classification in observational studies

Category	Criteria
**Convincing, class I**	● No. of cases >1000 or (more than 20000 participants for continuous outcomes)<br>● $P < 1\times10^{-6}$<br>● I^2 <50%<br>● 95% prediction intervals excluding the null value<br>● No small study effects<br>● No excess significance bias
**Highly suggestive, class II**	● No. of cases >1000 or (more than 20000 participants for continuous outcomes)<br>● $P < 1\times10^{-6}$<br>● The largest component study reporting a statistically significant result
**Suggestive, class III**	● No. of cases >1000 or (more than 20000 participants for continuous outcomes)<br>● $P < 1\times10^{-3}$
**Weak, class IV**	● $P < 0.05$
**Nonsignificant, V**	● $P > 0.05$

Table S6. Four economic regions in the mainland of China

Region	Covered provinces
**East China**	Beijing Municipality, Tianjin Municipality, Hebei province, Shanghai Municipality, Jiangsu province, Zhejiang province, Fujian province, Shandong province, Guangdong province, Hainan province
**Northeast China**	Liaoning province, Jilin province, Heilongjiang province
**Central China**	Shanxi province, Anhui province, Jiangxi province, Henan province, Hubei province, Hunan province
**West China**	Inner Mongolia Autonomous Region, Guangxi Zhuang Autonomous Region, Chongqing Municipality, Sichuan province, Guizhou province, Yunnan province, Tibet Autonomous Region, Shaanxi province, Gansu province, Qinghai province, Ningxia Hui Autonomous Region, Xinjiang Uyghur Autonomous Region

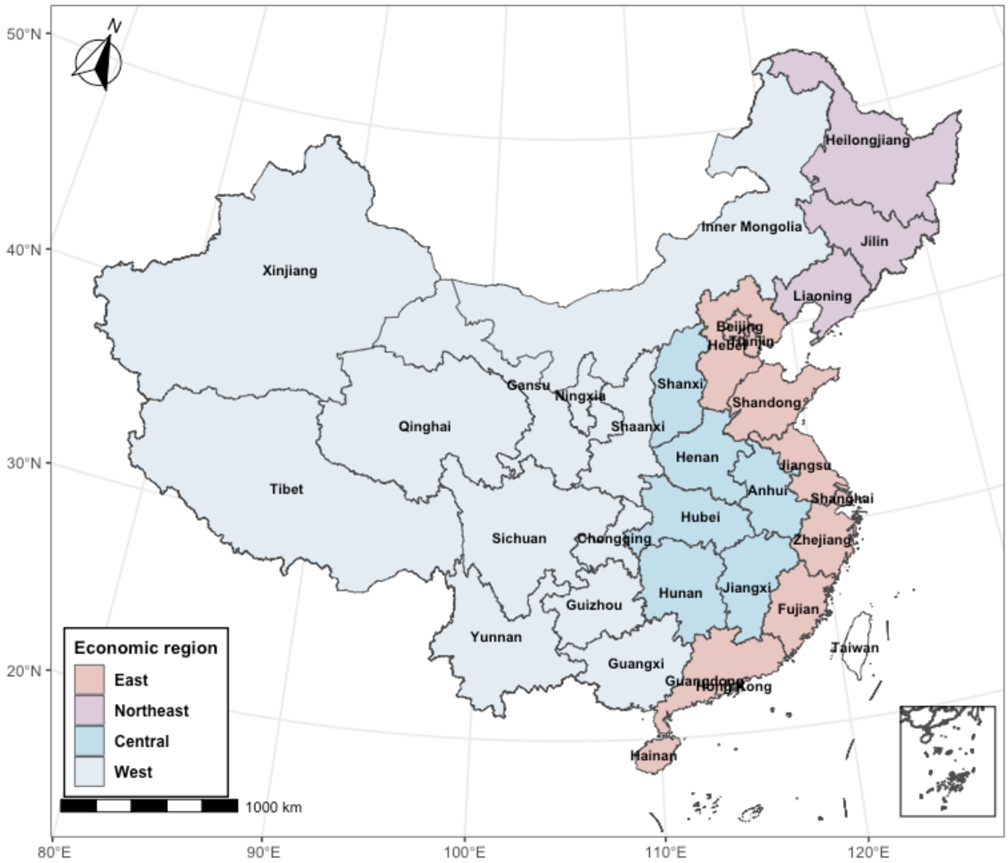

Table S7. Summary of the main characteristics of the included studies (N=54)

Characteristics	Number of studies (N=54, %)
**Year published**	
2005-2010	18 (33.33)
2011-2020	28 (51.85)
2021-2025	8 (14.81)
**Study design**	
Cross-sectional study	50 (92.59)
Cohort study	4 (7.41)
**Setting**	
Urban	23 (42.59)
Rural	13 (24.07)
Mixed	18 (33.33)
**Sample size**	
<1500	17 (31.48)
1500-3000	13 (24.08)
3001-5000	10 (18.52)
>5000	14 (25.93)
**Quality score**	
5	1 (1.85)
6	9 (9.26)
7	11 (20.37)
8	21 (14.81)
9	12 (16.67)

Table S8. Detailed characteristics of the included articles (N=54)

Study ID	Author (s)	Year published	Location	Study setting	Study year	Sampling	Study design	Age range (Year)	Sample size	Cases	Female proportion
**P-01**	Liu Chengguo, et al.	2005	Zhejiang, Zhoushan	Rural	2004	Cluster sampling	Cross-sectional	35-89	2668	57	0.4921
**P-02**	Chuang Shao-Yuan, et al.	2005	Taiwan, Kinmen	Rural	2002	Cluster sampling	Cross-sectional	40-79	1329	29	0.5418
**P-03**	Zheng Liqiang, et al.	2006	Sichuan, Panzhihua	Rural	2005	Random cluster sampling	Cross-sectional	18-85	1233	115	0.6334
**P-04**	He Yao, et al.	2006	Beijing	Urban	2001	Stratified sampling	Cross-sectional	60+	2334	198	0.5960
**P-05**	J. Woo, et al.	2006	Hong Kong	Urban	2006	Stratified sampling	Cross-sectional	65+	3998	274	0.5000
**P-06**	Liu Hao, et al.	2007	Sichuan, Panzhihua	Urban	2005	NA	Cross-sectional	40+	643	64	0.6003
**P-07**	Wang Linglin, et al.	2007	Shanghai	Urban	2006	Cluster sampling	Cross-sectional	60-97	2360	205	0.5225
**P-08**	Samuel Y.S. Wong, et al.	2007	Hong Kong	Urban	2002	Convenience stratified sampling	Cross-sectional	65+	3985	272	0.5019
**P-09**	Wang Yong, et al.	2009	Beijing, Shanghai, Changsha, Guangdong, Inner Mongolia, Xinjiang	Urban	2008	Stratified random cluster sampling	Cross-sectional	18+	21152	652	0.4865
**P-10**	Zhou Yueying, et al.	2009	Guangdong, Foshan	Rural	2008	Random cluster sampling	Cross-sectional	60-93	1447	165	0.5833
**P-11**	Samuel Y.S. Wong, et al.	2009	Hong Kong	Urban	NA	Stratified sampling	Cross-sectional	65+	1561	95	0.0000
**P-12**	Chen Jinghua, et al.	2010	Guangdong, Foshan	Rural	NA	Random cluster sampling	Cross-sectional	60-93	1679	205	0.5813
**P-13**	An Wei, et al.	2010	11 provinces	Mixed	NA	Random cluster sampling	Cross-sectional	35+	15817	981	0.5538
**P-14**	Zhang Xu, et al.	2010	Beijing	Urban	2009	Random sampling	Cross-sectional	60-94	988	127	0.6356
**P-15**	Yang Xiaomin, et al.	2010	Inner Mongolia, Baotou	Mixed	2009	Regular health examination	Cross-sectional	40-89	1040	138	0.4038
**P-16**	Wang Guipeng, et al.	2010	Xinjiang, Urumqi	Urban	2008	Random cluster sampling	Cross-sectional	60-93	1046	255	0.5793
**P-17**	Xu Songqing, et al.	2010	Zhejiang, Jiaxing	Urban	2008	Regular health examination	Cross-sectional	26-81	2994	73	0.6620
**P-18**	Xie Xiaoliang, et al.	2010	Shanghai, Inner Mongolia	Mixed	2007	Stratified random cluster sampling	Cross-sectional	15.3-93.5	2569	80	0.6123
**P-19**	Xiang Yang, et al.	2011	Xinjiang, Altay	Rural	2010	Stratified random cluster sampling	Cross-sectional	35-84	2082	97	0.4534
**P-20**	Zhou Lin, et al.	2012	Shanghai	Urban	2010	Cluster sampling	Cross-sectional	40+	5435	386	0.0403
**P-21**	Bai Xueqin, et al.	2012	Beijing	Urban	2008	Random sampling	Cross-sectional	20-96	7819	136	0.2463
**P-22**	J. Woo, et al.	2012	Hong Kong	Urban	2001	Stratified sampling	Cross-sectional	65+	3798	262	0.5153
**P-23**	Chen Ping.	2012	Xinjiang	Mixed	2009	Stratified random cluster sampling	Cross-sectional	35-84	8389	542	0.5488
**P-24**	Liu Lu.	2013	Guizhou	Mixed		Random cluster sampling	Cross-sectional	40+	4333	149	0.7307

Study ID	Author (s)	Year published	Location	Study setting	Study year	Sampling	Study design	Age range (Year)	Sample size	Cases	Female proportion
**P-25**	Zhu Hongmin.	2013	Shanghai	Rural	NA	Cluster sampling	Cross-sectional	65+	1492	51	0.6019
**P-26**	Wang Lanying, et al.	2013	Jiangsu, Xuzhou	Urban	2010	Cluster sampling	Cross-sectional	35+	1022	47	0.6536
**P-27**	Hu Bang-Chuan, et al.	2013	Shanghai	Rural	2006	Cluster sampling	Cross-sectional	NA	951	31	0.5058
**P-28**	Liu Yuping, et al.	2014	Sichuan, Chengdu	Mixed	2011	Random sampling	Cross-sectional	45+	6563	128	0.4030
**P-29**	Liang Yajun, et al.	2014	Shandong, Qufu	Rural	2011	Cluster sampling	Cross-sectional	NA	1499	85	0.5897
**P-30**	Yang Jinghui.	2014	Beijing	Urban	2010	Stratified random cluster sampling	Cross-sectional	60+	2101	147	0.5964
**P-31**	Lin Liming, et al.	2014	Hebei, Kailuan	Urban	2009	Stratified random sampling	Cross-sectional	40+	5184	182	0.3993
**P-32**	Han Hongfeng, et al.	2014	Hebei, Kailuan	Urban	2011	Regular health examination	Cross-sectional	NA	2408	113	0.5345
**P-33**	Pan Xinhua, et al.	2015	Zhejiang, Quzhou	Mixed	NA	Stratified cluster sampling	Cross-sectional	30-74	1730	234	0.6243
**P-34**	Lin Chih-Hsueh, et al.	2015	Taiwan, Taichung	Urban	2009	Cluster sampling	Cross-sectional	65+	1036	74	0.4797
**P-35**	Wen Jiangping, et al.	2015	Hebei, Yongnian	Rural	2007	Cluster sampling	Cohort	30+	4748	212	0.5482
**P-36**	Wang Anxin, et al.	2016	Hebei, Kailuan	Urban	2011	Stratified random sampling	Cross-sectional	40+	3048	161	0.4334
**P-37**	Huang Shan.	2016	Guangxi, Guilin, Liuzhou	Rural	NA	Stratified random sampling	Cross-sectional	NA	1283	43	0.7030
**P-38**	Wang Po, et al.	2017	Shanghai	Urban	2009	Stratified sampling	Cross-sectional	40+	3148	91	0.5972
**P-39**	Lu Liya, et al.	2017	Guangdong, Guangzhou	Mixed	2006	Random sampling	Cohort	50+	1507	24	0.6158
**P-40**	Mao Yong, et al.	2017	Beijing	Mixed	2008	Random sampling	Cohort	50+	4201	357	0.6365
**P-41**	Fan Xiaohong, et al.	2017	Beijing	Rural	2008	Stratified random cluster sampling	Cross-sectional	35+	9699	372	0.5290
**P-42**	Wang Zengwu, et al.	2018	Multiple provinces	Mixed	2014	Random sampling	Cross-sectional	35+	30025	2006	0.5423
**P-43**	Pan Jing, et al.	2018	Guangdong, Guangzhou	Mixed	2006	Stratified sampling	Cross-sectional	Mean age: 58.8	1395	23	0.5072
**P-44**	Zhao Qiannan, et al.	2019	Beijing	Mixed	2012	Stratified random sampling	Cross-sectional	35+	5208	258	0.5553
**P-45**	Cao Jingjing.	2019	Jiangsu, Anhui	Rural	2013	Cluster sampling	Cross-sectional	Mean age: 65.2	4009	115	0.6979
**P-46**	Wang Dandan, et al.	2020	Hebei	Mixed	2018	Cluster sampling	Cohort	Mean age: 57.1	3916	214	0.4231
**P-47**	Huang Shujing, et al.	2021	Jiangsu, Changzhou	Urban	2018	Cluster sampling	Cross-sectional	18+	5130	531	0.5870
**P-48**	Zheng Yue, et al.	2023	Shanghai	Mixed	2020	Stratified random sampling	Cross-sectional	60+	1293	106	0.5824
**P-49**	Wang Yong, et al.	2023	Shanghai	Mixed	2016	Stratified random sampling	Cross-sectional	18+	13144	256	0.4537
**P-50**	Wang Yaxing, et al.	2023	Beijing	Mixed	2011	Stratified random sampling	Cross-sectional	50+	1078	32	0.5900
**P-51**	Fu Xiya, et al.	2023	Shanghai	Mixed	2020	Stratified random sampling	Cross-sectional	65+	1357	125	0.5859

Study ID	Author (s)	Year published	Location	Study setting	Study year	Sampling	Study design	Age range (Year)	Sample size	Cases	Female proportion
**P-52**	Song Xiaohui, et al.	2023	Hubei, Beijing	Urban	2017	NA	Cross-sectional	18+	101465	2516	0.3472
**P-53**	Guo Qianlan.	2023	Jiangsu, Changzhou	Mixed	2018	Stratified random sampling	Cross-sectional	35+	2400	236	1.0000
**P-54**	Shang Qinggang, et al.	2024	Guangdong, Shenzhen	Urban	2021	Stratified random sampling	Cross-sectional	18+	6230	387	0.5027

Note: NA, not available.

Table S9. Included studies in meta-analysis of associated factors of peripheral artery disease in China

Factor	Author	Year Published	Sample size	Forest plot	Sensitivity analysis	Funnel plot
**Factor 1-Age (per one year increase)**						
	Liu Hao, et al.	2007	643	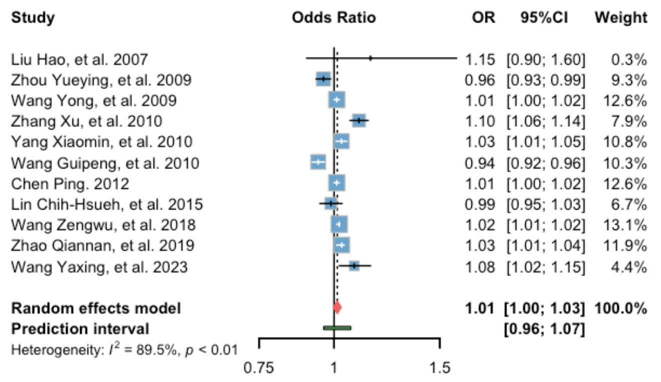	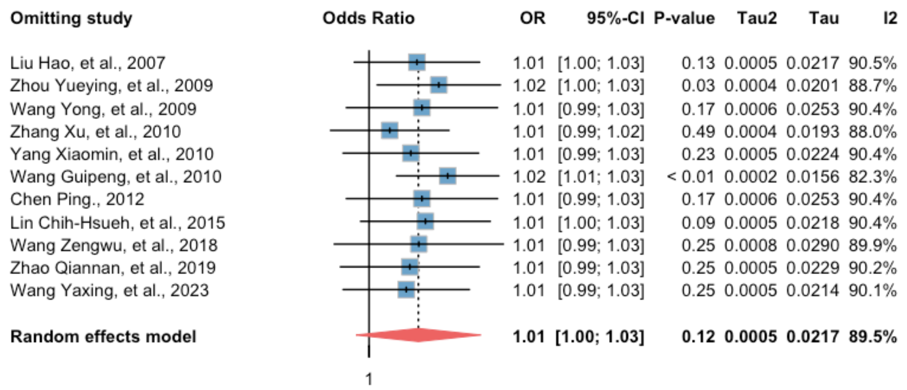	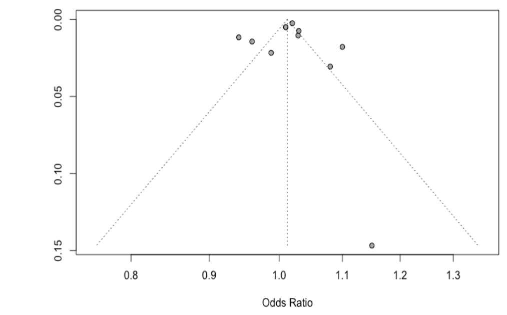
	Zhou Yueying, et al.	2009	1447			
	Wang Yong, et al.	2009	21539			
	Zhang Xu, et al.	2010	988			
	Yang Xiaomin, et al.	2010	1040			
	Wang Guipeng, et al.	2010	1046			
	Chen Ping.	2012	8389			
	Lin Chih-Hsueh, et al.	2015	1036			
	Wang Zengwu, et al.	2018	30025			
	Zhao Qiannan, et al.	2019	5208			
	Wang Yaxing, et al.	2023	1078			
**Factor 2-Sex-Female vs. Male**						
	Liu Hao, et al.	2007	643	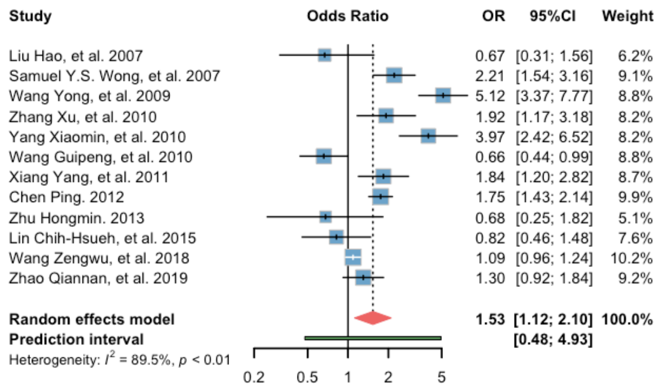	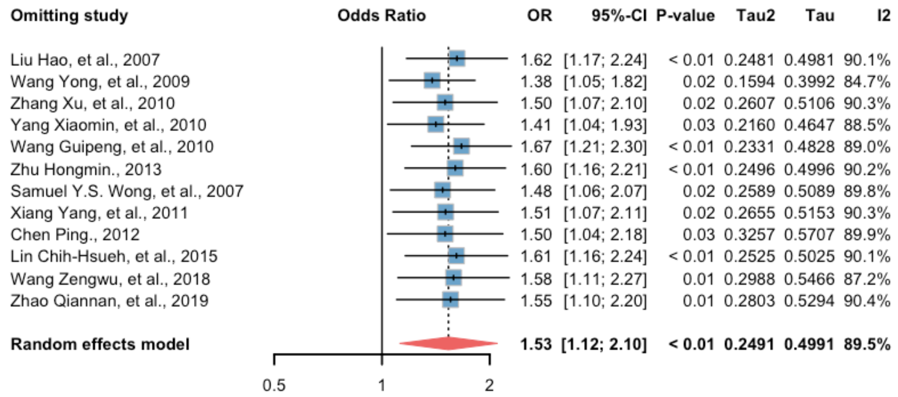	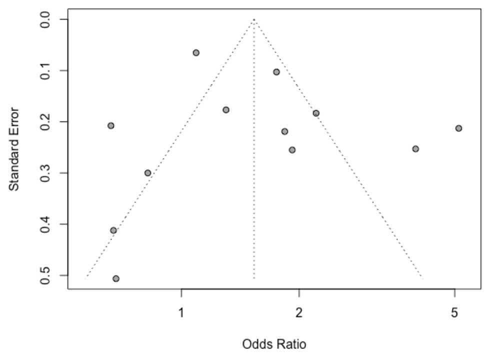
	Samuel Y.S. Wong, et al.	2007	3985			
	Wang Yong, et al.	2009	21539			
	Zhang Xu, et al.	2010	988			
	Yang Xiaomin, et al.	2010	1040			
	Wang Guipeng, et al.	2010	1046			
	Xiang Yang, et al.	2011	2082			
	Chen Ping.	2012	8389			
	Zhu Hongmin.	2013	1492			
	Lin Chih-Hsueh, et al.	2015	1036			
	Wang Zengwu, et al.	2018	30025			
	Zhao Qiannan, et al.	2019	5208			
**Factor 3-SBP (per ten mmHg increase)**						
	Zhou Yueying, et al.	2009	1447	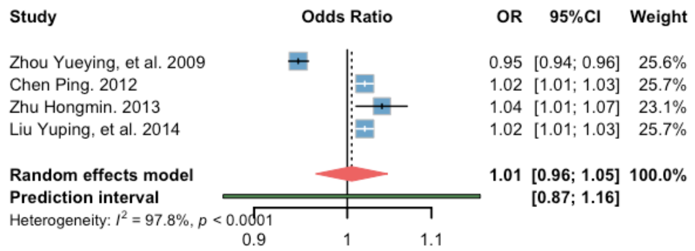	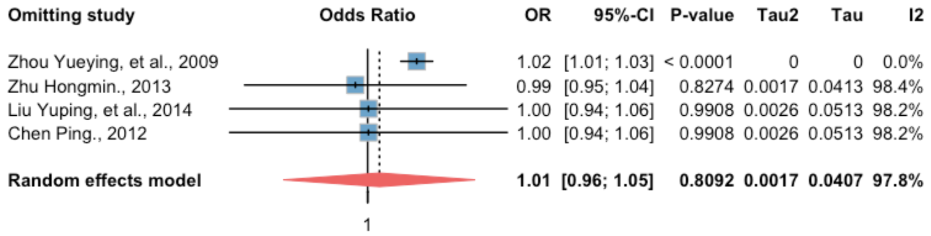	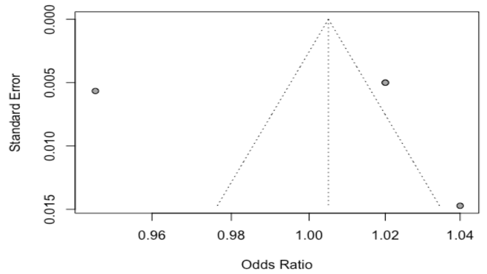
	Chen Ping.	2012	8389			
	Zhu Hongmin.	2013	1492			
	Liu Yuping, et al.	2014	6563			
**Factor 4-DBP (per ten mmHg increase)**						
	Chen Ping.	2012	8389	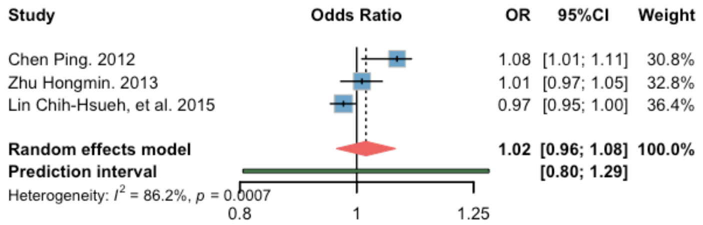	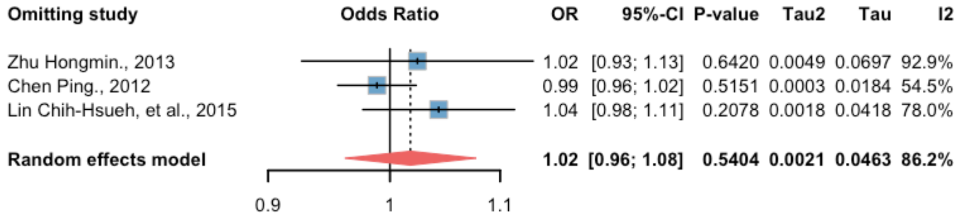	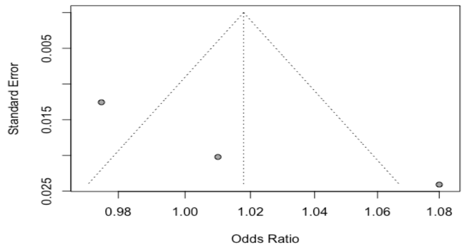
	Zhu Hongmin.	2013	1492			
	Lin Chih-Hsueh, et al.	2015	1036			
**Factor 5-BMI (per one kg/m² increase)**						
	Liu Hao, et al.	2007	643	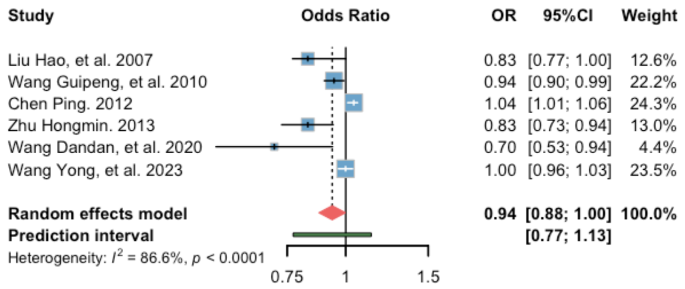	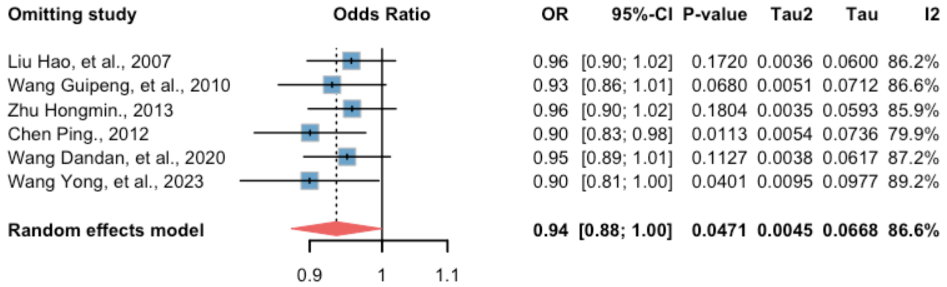	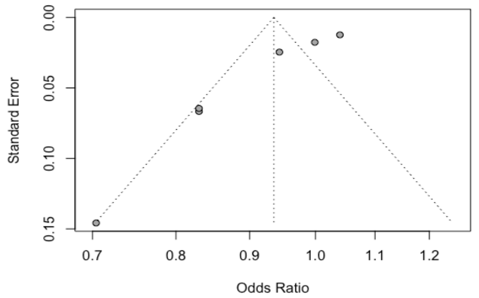
	Wang Guipeng, et al.	2010	1046			
	Chen Ping.	2012	8389			
	Zhu Hongmin.	2013	1492			
	Wang Dandan, et al.	2020	3916			
	Wang Yong, et al.	2023	13144			

Factor 6-WC (per one cm increase)

Factor	Author	Year Published	Sample size
	Zhou Yueying, et al.	2009	1447
	Wang Yong, et al.	2009	21539
	Wang Yong, et al.	2023	13144

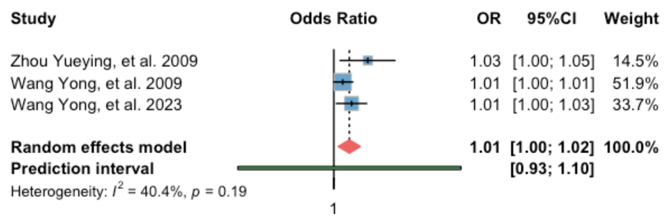

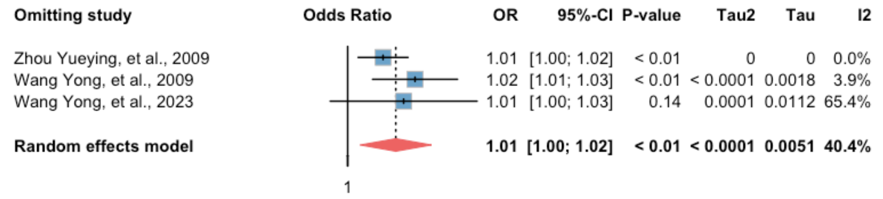

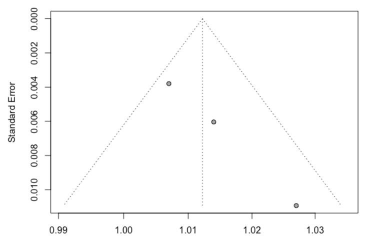

Factor 7-Current smoking-Yes vs. No

Factor	Author	Year Published	Sample size
	He Yao, et al.	2006	2334
	Liu Hao, et al.	2007	643
	Wang Yong, et al.	2009	21539
	Wang Guipeng, et al.	2010	1046
	Zhu Hongmin.	2013	1492
	Wang Zengwu, et al.	2018	30025
	Zhao Qiannan, et al.	2019	5208

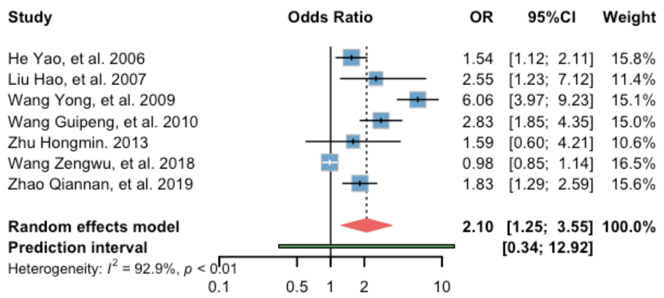

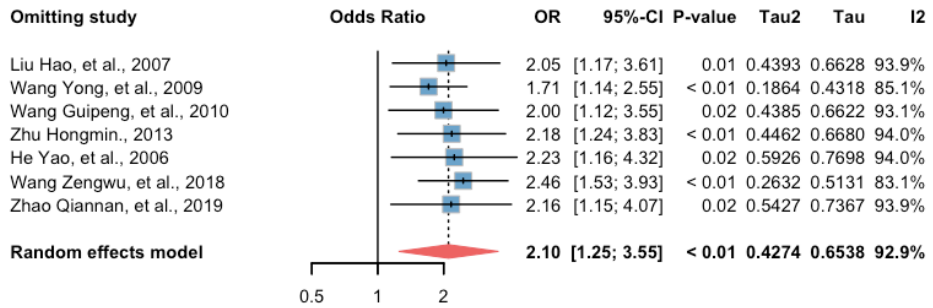

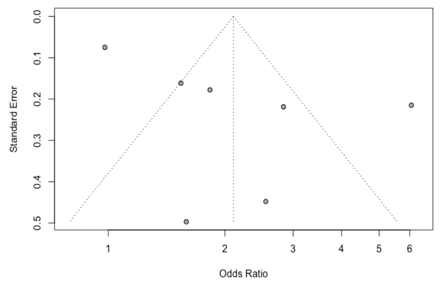

Factor 8-Former smoking-Yes vs. No

Factor	Author	Year Published	Sample size
	He Yao, et al.	2006	2334
	Samuel Y.S. Wong, et al.	2007	3985
	Wang Zengwu, et al.	2018	30025

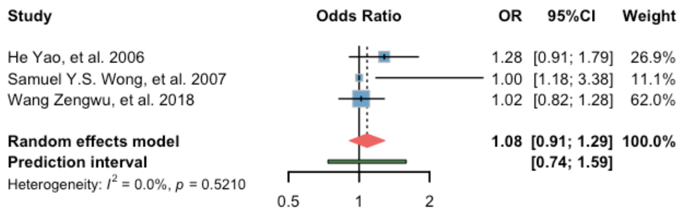

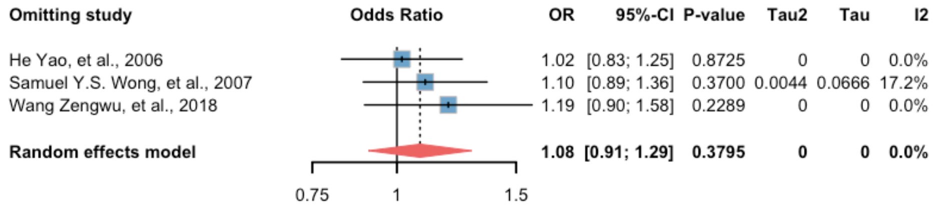

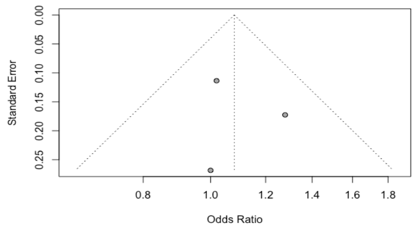

Factor 9-Current drinking-Yes vs. No

Factor	Author	Year Published	Sample size
	Liu Hao, et al.	2007	643
	Samuel Y.S. Wong, et al.	2007	3985
	Liang Yajun, et al.	2014	1499
	Wang Zengwu, et al.	2018	30025

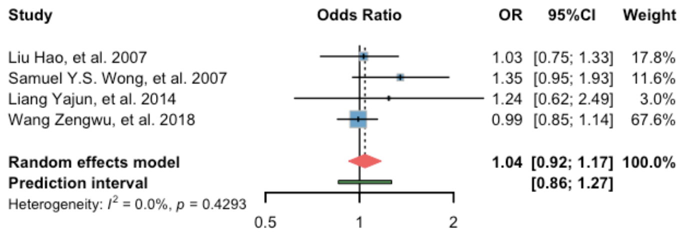

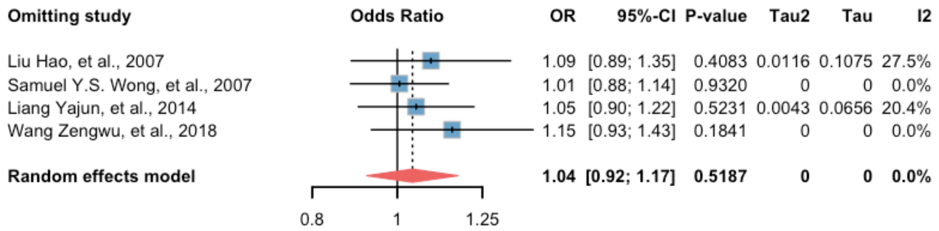

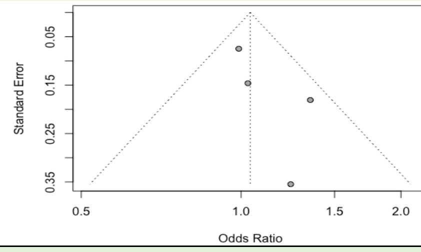

Factor 10-Hypertension-Yes vs. No

Factor	Author	Year Published	Sample size
	Liu Hao, et al.	2007	643
	Samuel Y.S. Wong, et al.	2007	3985
	Yang Xiaomin, et al.	2010	1040
	Wang Guipeng, et al.	2010	1046
	Xiang Yang, et al.	2011	2082
	Zhu Hongmin.	2013	1492
	Wang Zengwu, et al.	2018	30025
	Zhao Qiannan, et al.	2019	5208

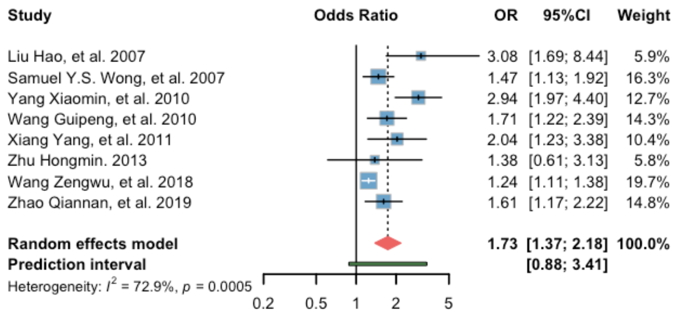

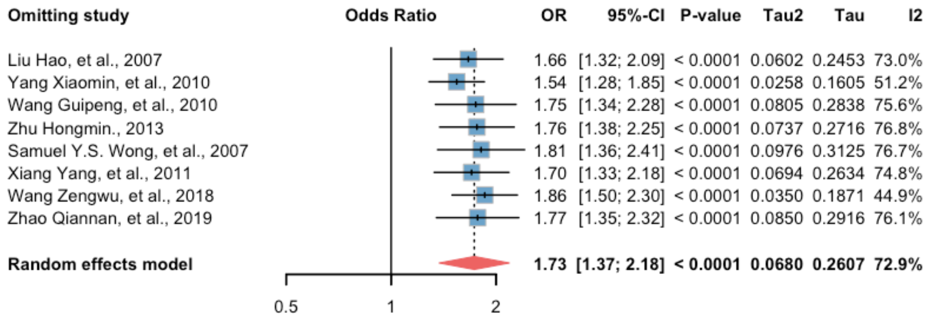

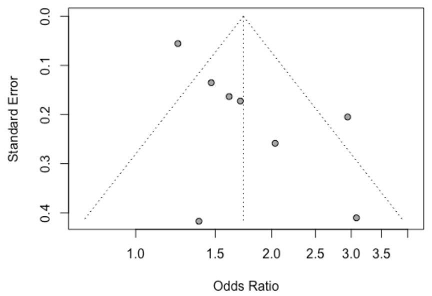

Factor 11-Diabetes-Yes vs. No

Factor	Author	Year Published	Sample size
	Liu Hao, et al.	2007	643
	Samuel Y.S. Wong, et al.	2007	3985
	Zhou Yueying, et al.	2009	1447
	Wang Yong, et al.	2009	21539
	Yang Xiaomin, et al.	2010	1040
	Chen Ping.	2012	8389
	Zhu Hongmin.	2013	1492
	Liang Yajun, et al.	2014	1499
	Wang Zengwu, et al.	2018	30025
	Zhao Qiannan, et al.	2019	5208

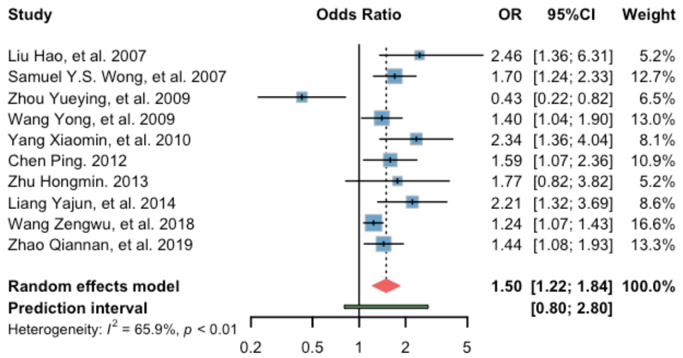

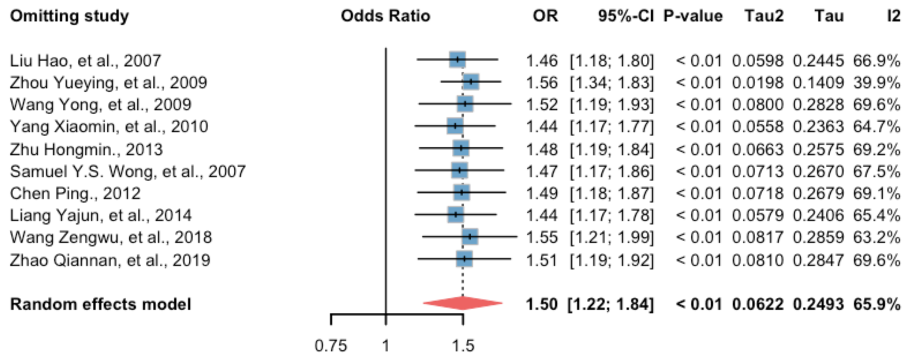

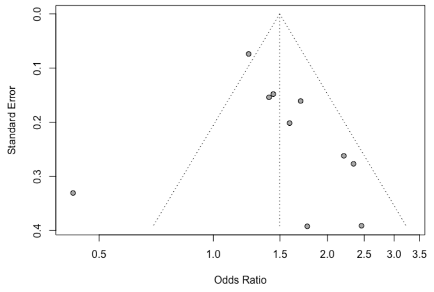

Factor	Author	Year Published	Sample size	Forest plot	Sensitivity analysis	Funnel plot
**Factor 12-Dyslipidemia-Yes vs. No**						
	Wang Yong, et al.	2009	21539	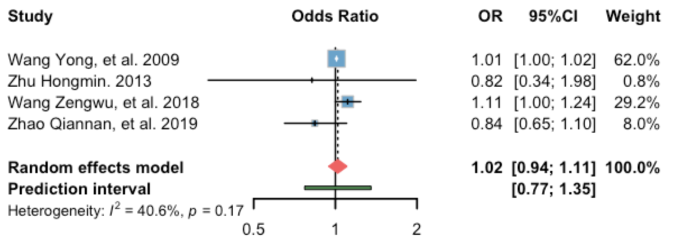	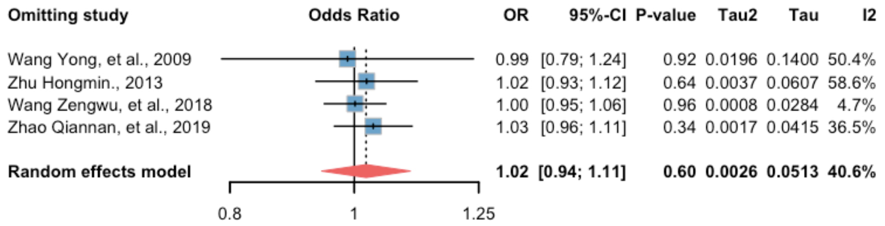	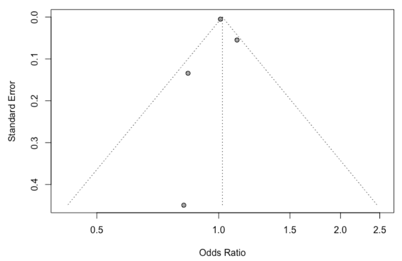
	Zhu Hongmin.	2013	1492			
	Wang Zengwu, et al.	2018	30025			
	Zhao Qiannan, et al.	2019	5208			
**Factor 13-CHD-Yes vs. No**						
	Wang Yong, et al.	2009	21539	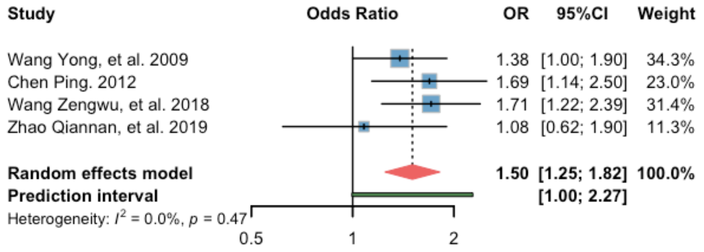	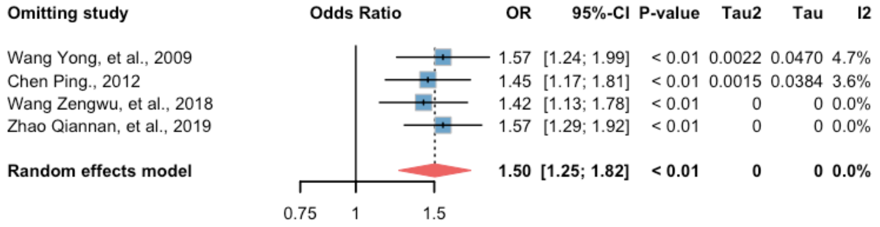	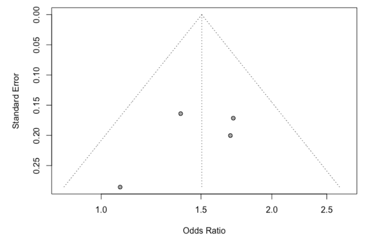
	Chen Ping.	2012	8389			
	Wang Zengwu, et al.	2018	30025			
	Zhao Qiannan, et al.	2019	5208			
**Factor 14-Stroke-Yes vs. No**						
	Wang Yong, et al.	2009	21539	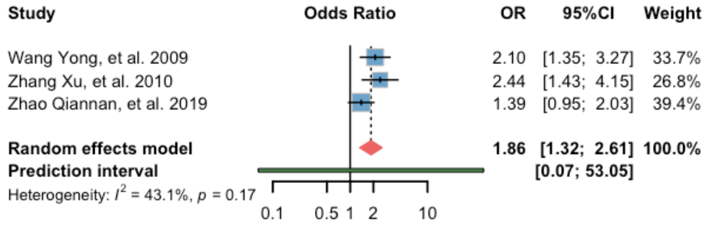	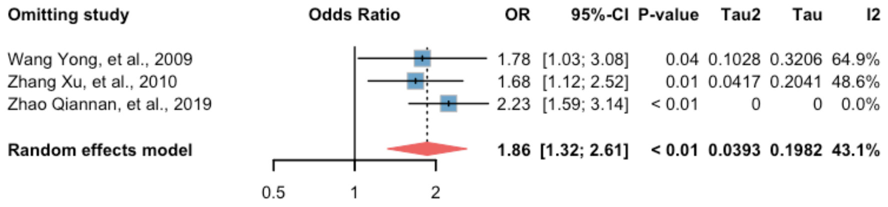	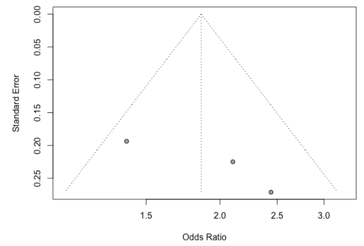
	Zhang Xu, et al.	2010	988			
	Zhao Qiannan, et al.	2019	5208			

Note: Synthesized effect size of 14 associated factors that were investigated in at least three studies using multivariate design. OR, odds ratio; CI, confidence interval; SBP, systolic blood pressure; DBP, diastolic blood pressure; BMI, body mass index; WC, waist circumference; CHD, coronary heart disease; I^2, I-square.

Table S10. Estimated age- and sex-specific prevalence and number of cases of peripheral artery disease by economic regions in the mainland of China in 2023

Age group in years	Prevalence of PAD, % (95%CI)			People living with PAD, million (95%CI)		
	Male	Female	Overall	Male	Female	Overall
**East**						
**30-34**	3.78 (3.19-4.47)	4.88 (4.13-5.76)	4.30 (3.64-5.09)	1.10 (0.92-1.30)	1.29 (1.09-1.52)	2.39 (2.02-2.82)
**35-39**	3.98 (3.36-4.70)	5.13 (4.34-6.05)	4.53 (3.83-5.35)	0.92 (0.78-1.09)	1.10 (0.93-1.30)	2.03 (1.71-2.39)
**40-44**	4.17 (3.53-4.93)	5.37 (4.54-6.33)	4.75 (4.02-5.61)	0.84 (0.71-0.99)	1.01 (0.85-1.19)	1.85 (1.56-2.18)
**45-49**	4.36 (3.69-5.15)	5.60 (4.74-6.60)	4.97 (4.20-5.86)	0.98 (0.82-1.15)	1.20 (1.02-1.42)	2.18 (1.84-2.57)
**50-54**	4.51 (3.81-5.33)	5.80 (4.91-6.83)	5.14 (4.35-6.07)	1.07 (0.91-1.27)	1.34 (1.13-1.58)	2.41 (2.04-2.85)
**55-59**	4.62 (3.91-5.46)	5.96 (5.05-7.02)	5.29 (4.48-6.24)	0.92 (0.78-1.09)	1.17 (0.99-1.38)	2.09 (1.77-2.47)
**60-64**	4.74 (4.01-5.59)	6.13 (5.19-7.21)	5.43 (4.60-6.40)	0.72 (0.61-0.85)	0.92 (0.78-1.08)	1.64 (1.39-1.93)
**65-69**	5.07 (4.29-5.98)	6.57 (5.57-7.73)	5.83 (4.94-6.87)	0.72 (0.61-0.85)	0.97 (0.82-1.14)	1.69 (1.44-2.00)
**70-74**	6.04 (5.12-7.12)	7.82 (6.65-9.18)	6.96 (5.91-8.18)	0.56 (0.48-0.66)	0.77 (0.65-0.90)	1.33 (1.13-1.56)
**75-79**	8.09 (6.88-9.49)	10.42 (8.90-12.18)	9.32 (7.95-10.91)	0.44 (0.37-0.52)	0.63 (0.54-0.74)	1.07 (0.92-1.26)
**80-84**	11.48 (9.82-13.38)	14.65 (12.59-16.98)	13.23 (11.35-15.37)	0.40 (0.34-0.47)	0.63 (0.55-0.74)	1.04 (0.89-1.21)
**85-89**	16.29 (14.04-18.81)	20.39 (17.69-23.39)	18.75 (16.23-21.56)	0.30 (0.26-0.35)	0.56 (0.49-0.65)	0.86 (0.75-0.99)
**Overall (30-89)**	**4.78 (4.04-5.63)**	**6.31 (5.36-7.42)**	**5.54 (4.70-6.52)**	**8.97 (7.59-10.58)**	**11.61 (9.86-13.64)**	**20.58 (17.45-24.22)**
**Northeast**						
**30-34**	3.63 (3.07-4.30)	5.36 (4.53-6.33)	4.48 (3.79-5.30)	0.15 (0.12-0.17)	0.21 (0.18-0.25)	0.36 (0.30-0.42)
**35-39**	3.81 (3.22-4.51)	5.61 (4.75-6.62)	4.70 (3.97-5.55)	0.13 (0.11-0.16)	0.19 (0.16-0.23)	0.33 (0.28-0.38)
**40-44**	3.99 (3.37-4.72)	5.90 (4.99-6.96)	4.93 (4.17-5.82)	0.15 (0.13-0.18)	0.22 (0.18-0.26)	0.37 (0.31-0.43)
**45-49**	4.16 (3.51-4.91)	6.16 (5.22-7.26)	5.15 (4.36-6.08)	0.19 (0.16-0.23)	0.28 (0.24-0.33)	0.47 (0.40-0.55)
**50-54**	4.35 (3.67-5.13)	6.41 (5.43-7.55)	5.37 (4.55-6.34)	0.22 (0.18-0.25)	0.32 (0.27-0.37)	0.53 (0.45-0.63)
**55-59**	4.50 (3.80-5.31)	6.56 (5.56-7.73)	5.53 (4.69-6.53)	0.21 (0.18-0.25)	0.31 (0.26-0.37)	0.52 (0.44-0.61)
**60-64**	4.65 (3.93-5.49)	6.70 (5.68-7.89)	5.70 (4.82-6.71)	0.18 (0.15-0.21)	0.27 (0.23-0.31)	0.45 (0.38-0.53)
**65-69**	5.00 (4.23-5.90)	7.21 (6.12-8.48)	6.15 (5.21-7.24)	0.17 (0.14-0.20)	0.26 (0.22-0.31)	0.42 (0.36-0.50)
**70-74**	5.96 (5.05-7.01)	8.61 (7.32-10.10)	7.37 (6.26-8.66)	0.12 (0.10-0.14)	0.19 (0.16-0.22)	0.31 (0.26-0.36)
**75-79**	7.99 (6.79-9.37)	11.48 (9.80-13.41)	9.90 (8.44-11.59)	0.09 (0.07-0.10)	0.15 (0.13-0.18)	0.24 (0.20-0.28)
**80-84**	11.24 (9.62-13.10)	16.08 (13.82-18.64)	13.96 (11.98-16.22)	0.08 (0.07-0.09)	0.15 (0.13-0.17)	0.23 (0.19-0.26)
**85-89**	15.96 (13.76-18.44)	22.41 (19.44-25.71)	19.65 (17.01-22.60)	0.05 (0.05-0.06)	0.10 (0.09-0.12)	0.16 (0.14-0.18)

Age group in years	Prevalence of PAD, % (95%CI)			People living with PAD, million (95%CI)		
	Male	Female	Overall	Male	Female	Overall
**Overall (30-89)**	**4.70 (3.98-5.54)**	**7.01 (5.95-8.24)**	**5.87 (4.98-6.91)**	**1.73 (1.47-2.04)**	**2.64 (2.24-3.10)**	**4.37 (3.71-5.15)**
**Central**						
**30-34**	3.60 (3.04-4.26)	4.51 (3.82-5.33)	4.06 (3.43-4.80)	0.53 (0.45-0.63)	0.67 (0.56-0.79)	1.19 (1.01-1.41)
**35-39**	3.79 (3.20-4.48)	4.72 (3.99-5.56)	4.25 (3.59-5.02)	0.45 (0.38-0.54)	0.56 (0.48-0.66)	1.02 (0.86-1.20)
**40-44**	3.97 (3.35-4.69)	4.94 (4.18-5.83)	4.45 (3.76-5.25)	0.49 (0.42-0.58)	0.61 (0.51-0.71)	1.10 (0.93-1.30)
**45-49**	4.15 (3.51-4.91)	5.19 (4.39-6.12)	4.66 (3.95-5.50)	0.62 (0.52-0.73)	0.75 (0.64-0.89)	1.37 (1.16-1.62)
**50-54**	4.33 (3.66-5.11)	5.39 (4.57-6.36)	4.86 (4.12-5.74)	0.66 (0.56-0.78)	0.83 (0.70-0.98)	1.49 (1.26-1.75)
**55-59**	4.45 (3.77-5.26)	5.52 (4.68-6.50)	4.99 (4.23-5.89)	0.57 (0.48-0.67)	0.72 (0.61-0.84)	1.29 (1.09-1.52)
**60-64**	4.60 (3.89-5.43)	5.64 (4.78-6.64)	5.11 (4.33-6.03)	0.41 (0.35-0.48)	0.49 (0.41-0.57)	0.90 (0.76-1.06)
**65-69**	4.93 (4.17-5.81)	6.08 (5.15-7.15)	5.51 (4.67-6.49)	0.45 (0.38-0.53)	0.56 (0.47-0.66)	1.00 (0.85-1.18)
**70-74**	5.87 (4.98-6.91)	7.24 (6.16-8.50)	6.56 (5.57-7.72)	0.37 (0.31-0.44)	0.47 (0.40-0.55)	0.84 (0.71-0.98)
**75-79**	7.81 (6.65-9.16)	9.66 (8.25-11.29)	8.78 (7.48-10.27)	0.30 (0.26-0.36)	0.41 (0.35-0.48)	0.72 (0.61-0.84)
**80-84**	11.04 (9.44-12.86)	13.52 (11.62-15.67)	12.41 (10.64-14.41)	0.25 (0.22-0.30)	0.38 (0.33-0.44)	0.64 (0.55-0.74)
**85-89**	15.60 (13.45-18.02)	18.87 (16.37-21.65)	17.56 (15.20-20.20)	0.17 (0.14-0.19)	0.30 (0.26-0.35)	0.47 (0.40-0.54)
**Overall (30-89)**	**4.64 (3.93-5.48)**	**5.87 (4.99-6.90)**	**5.26 (4.46-6.19)**	**5.27 (4.46-6.22)**	**6.74 (5.72-7.92)**	**12.01 (10.19-14.14)**
**West**						
**30-34**	3.78 (3.19-4.47)	4.63 (3.92-5.47)	4.19 (3.54-4.95)	0.61 (0.51-0.72)	0.70 (0.59-0.83)	1.31 (1.10-1.54)
**35-39**	3.95 (3.34-4.67)	4.86 (4.11-5.73)	4.39 (3.71-5.18)	0.56 (0.47-0.66)	0.64 (0.54-0.75)	1.20 (1.01-1.41)
**40-44**	4.17 (3.52-4.93)	5.12 (4.33-6.04)	4.63 (3.91-5.46)	0.56 (0.47-0.66)	0.65 (0.55-0.76)	1.21 (1.02-1.43)
**45-49**	4.38 (3.70-5.18)	5.37 (4.55-6.33)	4.86 (4.11-5.74)	0.73 (0.61-0.86)	0.85 (0.72-1.00)	1.58 (1.33-1.86)
**50-54**	4.56 (3.85-5.38)	5.55 (4.70-6.54)	5.05 (4.27-5.95)	0.71 (0.60-0.84)	0.84 (0.71-0.99)	1.55 (1.31-1.82)
**55-59**	4.68 (3.96-5.53)	5.68 (4.81-6.69)	5.18 (4.38-6.10)	0.59 (0.50-0.70)	0.70 (0.60-0.83)	1.29 (1.10-1.53)
**60-64**	4.81 (4.07-5.68)	5.80 (4.92-6.83)	5.30 (4.49-6.25)	0.43 (0.36-0.50)	0.50 (0.42-0.59)	0.93 (0.79-1.09)
**65-69**	5.20 (4.40-6.13)	6.26 (5.31-7.37)	5.74 (4.86-6.76)	0.47 (0.40-0.56)	0.59 (0.50-0.69)	1.06 (0.90-1.25)
**70-74**	6.21 (5.26-7.31)	7.48 (6.35-8.78)	6.85 (5.82-8.05)	0.39 (0.33-0.46)	0.49 (0.41-0.57)	0.88 (0.75-1.03)
**75-79**	8.30 (7.06-9.73)	9.96 (8.50-11.64)	9.17 (7.81-10.73)	0.35 (0.29-0.41)	0.46 (0.39-0.53)	0.80 (0.68-0.94)
**80-84**	11.70 (10.01-13.64)	13.94 (11.98-16.17)	12.92 (11.08-15.01)	0.29 (0.24-0.33)	0.41 (0.35-0.47)	0.69 (0.59-0.80)
**85-89**	16.45 (14.18-19.01)	19.37 (16.80-22.22)	18.13 (15.69-20.86)	0.17 (0.15-0.20)	0.28 (0.24-0.32)	0.45 (0.39-0.52)

**Age group in years**	**Prevalence of PAD, % (95%CI)**			**People living with PAD, million (95%CI)**		
	**Male**	**Female**	**Overall**	**Male**	**Female**	**Overall**
**Overall (30-89)**	**4.86 (4.12-5.73)**	**6.03 (5.12-7.08)**	**5.44 (4.61-6.40)**	**5.85 (4.95-6.90)**	**7.09 (6.02-8.33)**	**12.94 (10.97-15.23)**

Note: CI, confidence interval; PAD, peripheral artery disease.

Table S11. Estimated provincial prevalence and number of cases of peripheral artery disease in the mainland of China in 2023

Province	Prevalence of PAD, % (95%CI)			People living with PAD, million (95%CI)		
	Male	Female	Overall	Male	Female	Overall
Beijing	4.89 (4.14-5.76)	7.95 (6.75-9.34)	6.40 (5.43-7.53)	0.38 (0.32-0.45)	0.61 (0.52-0.71)	0.99 (0.84-1.16)
Tianjin	4.70 (3.98-5.54)	8.04 (6.83-9.46)	6.35 (5.38-7.47)	0.23 (0.19-0.27)	0.38 (0.32-0.45)	0.61 (0.52-0.72)
Hebei	4.11 (3.48-4.84)	6.32 (5.37-7.43)	5.23 (4.43-6.15)	0.99 (0.84-1.17)	1.55 (1.32-1.83)	2.54 (2.16-2.99)
Shanghai	4.89 (4.14-5.77)	7.16 (6.08-8.41)	6.00 (5.09-7.05)	0.45 (0.38-0.53)	0.63 (0.53-0.74)	1.08 (0.92-1.27)
Jiangsu	5.15 (4.37-6.07)	6.47 (5.50-7.60)	5.82 (4.94-6.84)	1.50 (1.27-1.77)	1.92 (1.63-2.25)	3.42 (2.91-4.03)
Zhejiang	5.02 (4.25-5.91)	6.17 (5.24-7.26)	5.58 (4.73-6.57)	1.15 (0.98-1.36)	1.34 (1.14-1.57)	2.49 (2.12-2.94)
Fujian	6.00 (5.08-7.08)	6.10 (5.18-7.17)	6.05 (5.13-7.12)	0.81 (0.68-0.95)	0.81 (0.68-0.95)	1.62 (1.37-1.90)
Shandong	4.05 (3.43-4.78)	5.93 (5.04-6.97)	5.00 (4.24-5.89)	1.37 (1.16-1.62)	2.05 (1.75-2.41)	3.43 (2.91-4.03)
Guangdong	4.99 (4.22-5.89)	5.99 (5.08-7.04)	5.47 (4.64-6.44)	1.95 (1.65-2.30)	2.15 (1.83-2.53)	4.10 (3.47-4.83)
Hainan	4.05 (3.43-4.78)	5.58 (4.74-6.55)	4.78 (4.06-5.63)	0.13 (0.11-0.15)	0.16 (0.14-0.19)	0.29 (0.25-0.34)
Liaoning	5.32 (4.51-6.27)	7.13 (6.06-8.38)	6.24 (5.29-7.34)	0.84 (0.72-1.00)	1.16 (0.99-1.37)	2.01 (1.70-2.36)
Jilin	4.18 (3.54-4.93)	7.42 (6.30-8.73)	5.82 (4.94-6.85)	0.37 (0.32-0.44)	0.68 (0.57-0.79)	1.05 (0.89-1.23)
Heilongjiang	4.27 (3.62-5.04)	6.54 (5.56-7.69)	5.42 (4.59-6.37)	0.51 (0.44-0.61)	0.80 (0.68-0.94)	1.32 (1.12-1.55)
Shanxi	4.71 (3.99-5.55)	6.49 (5.51-7.63)	5.59 (4.74-6.58)	0.55 (0.47-0.65)	0.74 (0.63-0.87)	1.29 (1.09-1.52)
Anhui	4.44 (3.76-5.23)	6.10 (5.18-7.16)	5.27 (4.47-6.20)	0.86 (0.73-1.01)	1.20 (1.02-1.41)	2.06 (1.75-2.42)
Jiangxi	4.62 (3.91-5.45)	5.43 (4.61-6.39)	5.03 (4.27-5.92)	0.63 (0.53-0.74)	0.74 (0.63-0.87)	1.37 (1.16-1.61)
Henan	4.78 (4.05-5.64)	6.16 (5.23-7.24)	5.49 (4.66-6.47)	1.39 (1.18-1.64)	1.90 (1.62-2.24)	3.29 (2.79-3.87)
Hubei	4.47 (3.78-5.27)	5.44 (4.62-6.40)	4.95 (4.19-5.83)	0.80 (0.68-0.95)	0.95 (0.81-1.12)	1.75 (1.49-2.07)
Hunan	4.77 (4.04-5.62)	5.56 (4.72-6.53)	5.16 (4.38-6.07)	1.04 (0.88-1.23)	1.21 (1.02-1.42)	2.25 (1.91-2.64)
Inner Mongolia	4.82 (4.08-5.68)	6.19 (5.25-7.28)	5.50 (4.66-6.47)	0.42 (0.36-0.50)	0.53 (0.45-0.62)	0.95 (0.80-1.12)
Guangxi	3.95 (3.35-4.66)	5.46 (4.64-6.41)	4.69 (3.98-5.52)	0.60 (0.51-0.71)	0.80 (0.68-0.94)	1.41 (1.19-1.66)
Chongqing	3.75 (3.17-4.41)	6.25 (5.31-7.35)	5.00 (4.25-5.88)	0.40 (0.34-0.47)	0.67 (0.57-0.78)	1.06 (0.90-1.25)
Sichuan	5.89 (4.99-6.94)	6.60 (5.61-7.75)	6.24 (5.30-7.34)	1.64 (1.39-1.94)	1.84 (1.57-2.17)	3.49 (2.96-4.11)
Guizhou	5.53 (4.68-6.52)	6.17 (5.24-7.25)	5.85 (4.96-6.88)	0.61 (0.51-0.72)	0.67 (0.57-0.79)	1.28 (1.08-1.50)
Yunnan	5.25 (4.45-6.20)	5.94 (5.04-6.99)	5.59 (4.74-6.58)	0.70 (0.59-0.83)	0.75 (0.64-0.88)	1.45 (1.23-1.71)
Tibet	3.28 (2.78-3.87)	5.54 (4.70-6.52)	4.35 (3.68-5.12)	0.03 (0.03-0.04)	0.05 (0.04-0.06)	0.08 (0.07-0.10)
Shaanxi	4.70 (3.98-5.54)	5.79 (4.92-6.81)	5.24 (4.44-6.17)	0.62 (0.52-0.73)	0.75 (0.64-0.88)	1.37 (1.16-1.61)

Province	Prevalence of PAD, % (95%CI)			People living with PAD, million (95%CI)		
	Male	Female	Overall	Male	Female	Overall
**Gansu**	4.54 (3.85-5.36)	5.56 (4.72-6.54)	5.05 (4.28-5.94)	0.36 (0.31-0.43)	0.44 (0.37-0.52)	0.80 (0.68-0.95)
**Qinghai**	4.37 (3.70-5.16)	5.28 (4.48-6.21)	4.81 (4.08-5.67)	0.08 (0.07-0.09)	0.09 (0.08-0.11)	0.17 (0.14-0.20)
**Ningxia**	5.09 (4.31-6.00)	5.58 (4.73-6.56)	5.33 (4.52-6.28)	0.11 (0.09-0.13)	0.12 (0.10-0.14)	0.23 (0.20-0.27)
**Xinjiang**	3.67 (3.11-4.34)	5.65 (4.79-6.65)	4.62 (3.91-5.45)	0.27 (0.23-0.31)	0.38 (0.32-0.44)	0.64 (0.54-0.76)

Note: CI, confidence interval; PAD, peripheral artery disease.

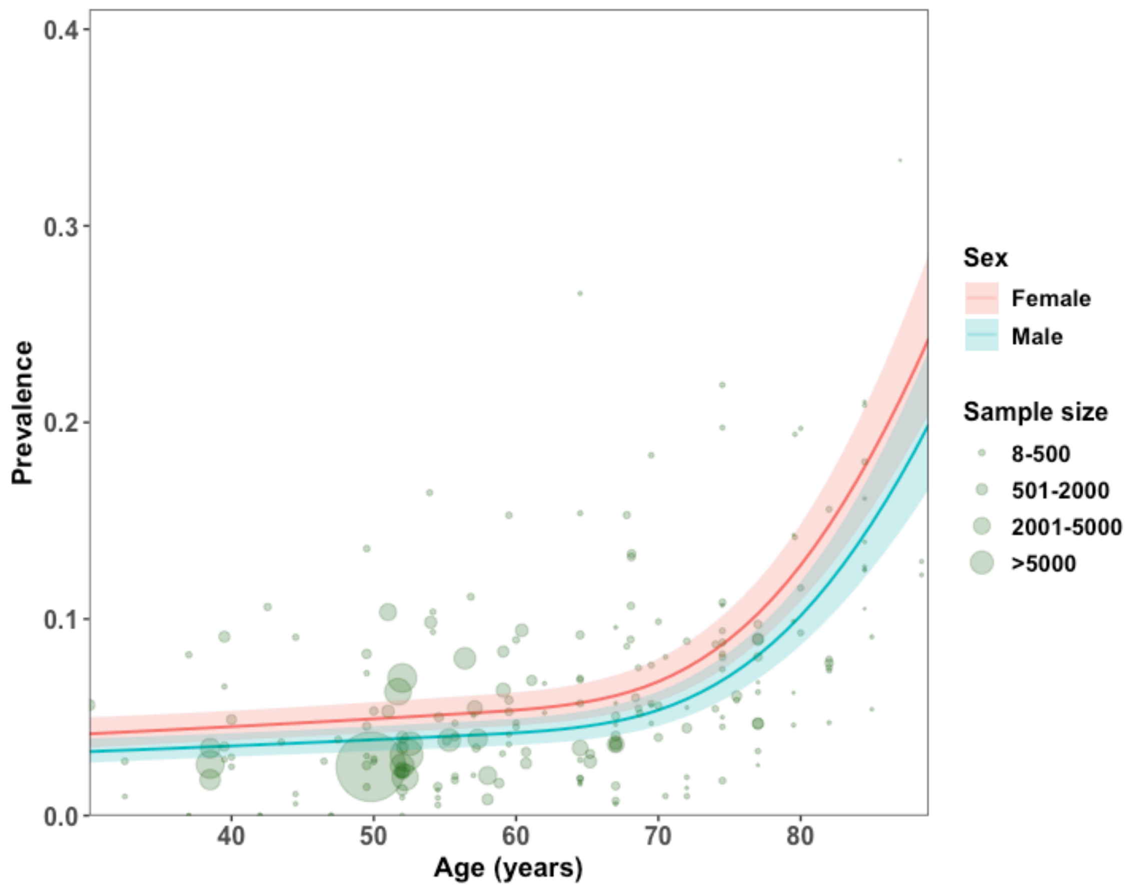

Figure S1. Rate pattern for peripheral artery disease prevalence in China

Figure S2. Quality scores of the included articles using the Joanna Briggs Institute Critical Appraisal Checklist for Prevalence Studies

Article ID	Author (s)	Year Published	Q1	Q2	Q3	Q4	Q5	Q6	Q7	Q8	Q9	Total
**P-01**	Liu Chengguo, et al.	2005	Yes	Yes	Yes	Yes	Yes	Yes	Yes	Yes	No	8
**P-02**	Chuang Shao-Yuan, et al.	2005	Yes	Yes	No	Yes	Yes	Yes	Unclear	Yes	No	6
**P-03**	Zheng Liqiang, et al.	2006	Yes	Yes	No	Yes	Yes	Yes	Yes	Yes	Unclear	7
**P-04**	He Yao, et al.	2006	Yes	Yes	Yes	Yes	Yes	Yes	Yes	Yes	Yes	9
**P-05**	J. Woo, et al.	2006	Yes	Yes	Yes	Yes	Yes	Yes	Yes	Yes	Yes	9
**P-06**	Liu Hao, et al.	2007	Yes	Unclear	No	Yes	Yes	Yes	Yes	Yes	Unclear	6
**P-07**	Wang Linglin, et al.	2007	Yes	Unclear	Yes	Yes	Yes	Unclear	Yes	Yes	Unclear	6
**P-08**	Samuel Y.S. Wong, et al.	2007	Yes	Yes	Yes	Yes	Yes	Yes	Unclear	Yes	Yes	8
**P-09**	Wang Yong, et al.	2009	Yes	Yes	Yes	Yes	Yes	Yes	Yes	Yes	No	8
**P-10**	Zhou Yueying, et al.	2009	Yes	Yes	No	Yes	Yes	Yes	Unclear	Yes	Yes	7
**P-11**	Samuel Y.S. Wong, et al.	2009	Yes	Yes	No	Yes	Yes	Yes	Unclear	Yes	Yes	7
**P-12**	Chen Jinghua, et al.	2010	Yes	Yes	Yes	Yes	Yes	Yes	Yes	Yes	No	8
**P-13**	An Wei, et al.	2010	Yes	Yes	Yes	Yes	Yes	Unclear	Unclear	Yes	Yes	7
**P-14**	Zhang Xu, et al.	2010	Yes	Yes	No	Yes	Yes	Yes	Unclear	Yes	Unclear	6
**P-15**	Yang Xiaomin, et al.	2010	Yes	No	No	Yes	Yes	Yes	Yes	Yes	Yes	7
**P-16**	Wang Guipeng, et al.	2010	Yes	Yes	No	Yes	Yes	Yes	Yes	Yes	Yes	8
**P-17**	Xu Songqing, et al.	2010	Yes	Yes	Yes	Yes	Yes	Yes	Yes	Yes	Unclear	8
**P-18**	Xie Xiaoliang, et al.	2010	Yes	Yes	Yes	Yes	Yes	Yes	Yes	Yes	Yes	9
**P-19**	Xiang Yang, et al.	2011	Yes	Yes	Yes	Yes	Yes	Yes	Yes	Yes	Yes	9
**P-20**	Zhou Lin, et al.	2012	Yes	Yes	Yes	Yes	Yes	Yes	Yes	Yes	Yes	**9**
**P-21**	Bai Xueqin, et al.	2012	Yes	No	Yes	Yes	Yes	Yes	Yes	Yes	Yes	8
**P-22**	J. Woo, et al.	2012	Yes	Yes	Yes	Yes	Yes	Yes	Unclear	Yes	Yes	8

Article ID	Author (s)	Year Published	Q1	Q2	Q3	Q4	Q5	Q6	Q7	Q8	Q9	Total
**P-23**	Chen Ping.	2012	Yes	Yes	Yes	Yes	Yes	Yes	Yes	Yes	Yes	**9**
**P-24**	Liu Lu.	2013	Yes	Yes	Yes	Yes	Yes	Unclear	Unclear	Yes	Unclear	**6**
**P-25**	Zhu Hongmin.	2013	Yes	Yes	No	Yes	Yes	Yes	Unclear	Yes	Unclear	**6**
**P-26**	Wang Lanying, et al.	2013	Yes	Yes	No	Yes	Yes	Unclear	Unclear	Yes	Unclear	**5**
**P-27**	Hu Bang-Chuan, et al.	2013	No	Yes	No	Yes	Yes	Yes	Yes	Yes	No	**6**
**P-28**	Liu Yuping, et al.	2014	Yes	Yes	Yes	Yes	Yes	Yes	Yes	Yes	Yes	**9**
**P-29**	Liang Yajun, et al.	2014	Yes	Yes	No	Yes	Yes	Yes	Yes	Yes	Yes	**8**
**P-30**	Yang Jinghui.	2014	Yes	Yes	Yes	Yes	Yes	Yes	Yes	Yes	Yes	**9**
**P-31**	Lin Liming, et al.	2014	Yes	Yes	Yes	Yes	Yes	Yes	Yes	Yes	Yes	**9**
**P-32**	Han Hongfeng, et al.	2014	Yes	No	Yes	Yes	Yes	Unclear	Yes	Yes	Yes	**7**
**P-33**	Pan Xinhua, et al.	2015	Yes	Yes	Yes	Yes	Yes	Yes	Unclear	Yes	Yes	**8**
**P-34**	Lin Chih-Hsueh, et al.	2015	Yes	Yes	No	Yes	Yes	Unclear	Unclear	Yes	Yes	**6**
**P-35**	Wen Jiangping, et al.	2015	Yes	Yes	Yes	Yes	Yes	Yes	Yes	Yes	No	**8**
**P-36**	Wang Anxin, et al.	2016	Yes	Yes	Yes	Yes	Yes	Yes	Yes	Yes	No	**8**
**P-37**	Huang Shan.	2016	Yes	Yes	No	Yes	Yes	Yes	Yes	Yes	Yes	**8**
**P-38**	Wang Po, et al.	2017	Yes	Yes	Yes	Yes	Yes	Yes	Yes	Yes	No	**8**
**P-39**	Lu Liya, et al.	2017	Yes	Yes	No	Yes	Yes	Yes	Yes	Yes	No	**7**
**P-40**	Mao Yong, et al.	2017	Yes	Yes	Yes	Yes	Yes	Yes	Yes	Yes	No	**8**
**P-41**	Fan Xiaohong, et al.	2017	Yes	Yes	Yes	Yes	Yes	Yes	Yes	Yes	No	**8**
**P-42**	Wang Zengwu, et al.	2018	Yes	Yes	Yes	Yes	Yes	Yes	Yes	Yes	No	**8**
**P-43**	Pan Jing, et al.	2018	Yes	Yes	No	Yes	Yes	Yes	Yes	Yes	No	**7**
**P-44**	Zhao Qiannan, et al.	2019	Yes	Yes	Yes	Yes	Yes	Yes	Yes	Yes	No	**8**
**P-45**	Cao Jingjing.	2019	Yes	Yes	Yes	Yes	Yes	Yes	Yes	Yes	No	**8**
**P-46**	Wang Dandan, et al.	2020	Yes	Yes	Yes	Yes	Yes	Yes	Yes	Yes	No	**8**

Article ID	Author (s)	Year Published	Q1	Q2	Q3	Q4	Q5	Q6	Q7	Q8	Q9	Total
**P-47**	Huang Shujing, et al.	2021	Yes	Yes	Yes	Yes	Yes	Yes	Yes	Yes	Yes	**9**
**P-48**	Zheng Yue, et al.	2023	Yes	Yes	No	Yes	Yes	Yes	Yes	Yes	Unclear	**7**
**P-49**	Wang Yong, et al.	2023	Yes	Yes	Yes	Yes	Yes	Yes	Yes	Yes	Yes	**9**
**P-50**	Wang Yaxing, et al.	2023	Yes	Yes	No	Yes	Yes	Yes	Yes	Yes	No	**7**
**P-51**	Fu Xiya, et al.	2023	Yes	Yes	No	Yes	Yes	Yes	Yes	Yes	Yes	**8**
**P-52**	Song Xiaohui, et al.	2023	Unclear	Unclear	Yes	Yes	Yes	Yes	Unclear	Yes	Yes	**6**
**P-53**	Guo Qianlan.	2023	No	Yes	Yes	Yes	Yes	Yes	Yes	Yes	No	**7**
**P-54**	Shang Qinggang, et al.	2024	Yes	Yes	Yes	Yes	Yes	Yes	Yes	Yes	Yes	**9**

Study	Location	Sample	Age range	Prevalence(%)	95% CI	Weight
Liu Chengguo, et al. 2005	Zhejiang, Zhoushan	2668	35-89	2.14	[1.62; 2.76]	1.9%
Chuang Shao-Yuan, et al. 2005	Taiwan, Kinmen	1329	40-79	2.18	[1.47; 3.12]	1.8%
Zheng Liqiang, et al. 2006	Sichuan, Panzhihua	1233	18-85	9.33	[7.76; 11.09]	1.8%
He Yao, et al. 2006	Beijing	2334	60+	8.48	[7.38; 9.69]	1.9%
J. Woo, et al. 2006	Hong Kong	3998	65+	6.85	[6.09; 7.68]	1.9%
Liu Hao, et al. 2007	Sichuan, Panzhihua	643	40+	9.95	[7.75; 12.53]	1.7%
Wang Linglin, et al. 2007	Shanghai	2360	60-97	8.69	[7.58; 9.90]	1.9%
Samuel Y.S. Wong, et al. 2007	Hong Kong	3985	65+	6.83	[6.06; 7.65]	1.9%
Wang Yong, et al. 2009	6 provinces	21152	18+	3.08	[2.85; 3.32]	1.9%
Zhou Yueying, et al. 2009	Guangdong, Foshan	1447	60-93	11.40	[9.81; 13.15]	1.8%
Samuel Y.S. Wong, et al. 2009	Hong Kong	1561	65+	6.09	[4.95; 7.39]	1.8%
Chen Jinghua, et al. 2010	Guangdong, Foshan	1679	60-93	12.21	[10.68; 13.87]	1.8%
An Wei, et al. 2010	11 provinces	15817	35+	6.20	[5.83; 6.59]	1.9%
Zhang Xu, et al. 2010	Beijing	988	60-94	12.85	[10.83; 15.10]	1.8%
Yang Xiaomin, et al. 2010	Inner Mongolia, Baotou	1040	40-89	13.27	[11.27; 15.48]	1.8%
Wang Guipeng, et al. 2010	Xinjiang, Urumqi	1046	60-93	24.38	[21.80; 27.10]	1.8%
Xu Songqing, et al. 2010	Zhejiang, Jiaxing	2994	26-81	2.44	[1.92; 3.06]	1.9%
Xie Xiaoliang, et al. 2010	Shanghai, Inner Mongolia	2569	15.3-93.5	3.11	[2.48; 3.86]	1.9%
Xiang Yang, et al. 2011	Xinjiang, Altay	2082	35-84	4.66	[3.79; 5.65]	1.9%
Zhou Lin, et al. 2012	Shanghai	5435	40+	7.10	[6.43; 7.82]	1.9%
Bai Xueqin, et al. 2012	Beijing	7819	20-96	1.74	[1.46; 2.05]	1.9%
J. Woo, et al. 2012	Hong Kong	3798	65+	6.90	[6.11; 7.75]	1.9%
Chen Ping. 2012	Xinjiang	8389	35-84	6.46	[5.94; 7.01]	1.9%
Liu Lu. 2013	Guizhou	4333	40+	3.44	[2.92; 4.03]	1.9%
Zhu Hongmin. 2013	Shanghai	1492	65+	3.42	[2.56; 4.47]	1.9%
Wang Lanying, et al. 2013	Jiangsu, Xuzhou	1022	35+	4.60	[3.40; 6.07]	1.8%
Hu Bang-Chuan, et al. 2013	Shanghai	951		3.26	[2.23; 4.60]	1.8%
Liu Yuping, et al. 2014	Sichuan, Chengdu	6563	45+	1.95	[1.63; 2.31]	1.9%
Liang Yajun, et al. 2014	Shandong, Qufu	1499		5.67	[4.55; 6.96]	1.8%
Yang Jinghui. 2014	Beijing	2101	60+	7.00	[5.94; 8.17]	1.9%
Lin Liming, et al. 2014	Hebei, Kailuan	5184	40+	3.51	[3.03; 4.05]	1.9%
Han Hongfeng, et al. 2014	Hebei, Kailuan	2408		4.69	[3.88; 5.61]	1.9%
Pan Xinhua, et al. 2015	Zhejiang, Quzhou	1730	30-74	13.53	[11.95; 15.23]	1.8%
Lin Chih-Hsueh, et al. 2015	Taiwan, Taichung	1036	65+	7.14	[5.65; 8.88]	1.8%
Wen Jiangping, et al. 2015	Hebei, Yongnian	4748	30+	4.47	[3.90; 5.09]	1.9%
Wang Anxin, et al. 2016	Hebei, Kailuan	3048	40+	5.28	[4.51; 6.14]	1.9%
Huang Shan. 2016	Guangxi, Guilin, Liuzhou	1283		3.35	[2.44; 4.49]	1.8%
Wang Po, et al. 2017	Shanghai	3148	40+	2.89	[2.33; 3.54]	1.9%
Lu Liya, et al. 2017	Guangdong, Guangzhou	1507	50+	1.59	[1.02; 2.36]	1.8%
Mao Yong, et al. 2017	Beijing	4201	50+	8.50	[7.67; 9.38]	1.9%
Fan Xiaohong, et al. 2017	Beijing	9699	35+	3.84	[3.46; 4.24]	1.9%
Wang Zengwu, et al. 2018	Multiple provinces	30025	35+	6.68	[6.40; 6.97]	1.9%
Pan Jing, et al. 2018	Guangdong, Guangzhou	1395	Mean age: 58.8	1.65	[1.05; 2.46]	1.8%
Zhao Qiannan, et al. 2019	Beijing	5208	35+	4.95	[4.38; 5.58]	1.9%
Cao Jingjing. 2019	Jiangsu, Anhui	4009	Mean age: 65.2	2.87	[2.37; 3.43]	1.9%
Wang Dandan, et al. 2020	Hebei	3916	Mean age: 57.1	5.46	[4.77; 6.22]	1.9%
Huang Shujing, et al. 2021	Jiangsu, Changzhou	5130	18+	10.35	[9.53; 11.22]	1.9%
Zheng Yue, et al. 2023	Shanghai	1293	60+	8.20	[6.76; 9.83]	1.8%
Wang Yong, et al. 2023	Shanghai	13144	18+	1.95	[1.72; 2.20]	1.9%
Wang Yaxing, et al. 2023	Beijing	1078	50+	2.97	[2.04; 4.16]	1.8%
Fu Xiya, et al. 2023	Shanghai	1357	65+	9.21	[7.73; 10.88]	1.8%
Song Xiaohui, et al. 2023	Hubei, Beijing	101465	18+	2.48	[2.38; 2.58]	1.9%
Guo Qianlan. 2023	Jiangsu, Changzhou	2400	35+	9.83	[8.67; 11.09]	1.9%
Shang Qinggang, et al. 2024	Guangdong, Shenzhen	6230	18+	6.21	[5.63; 6.84]	1.9%
**Random effects model**				**5.70**	**[4.93; 6.52]**	**100.0%**

Heterogeneity: I^2 = 98.9%, p = 0

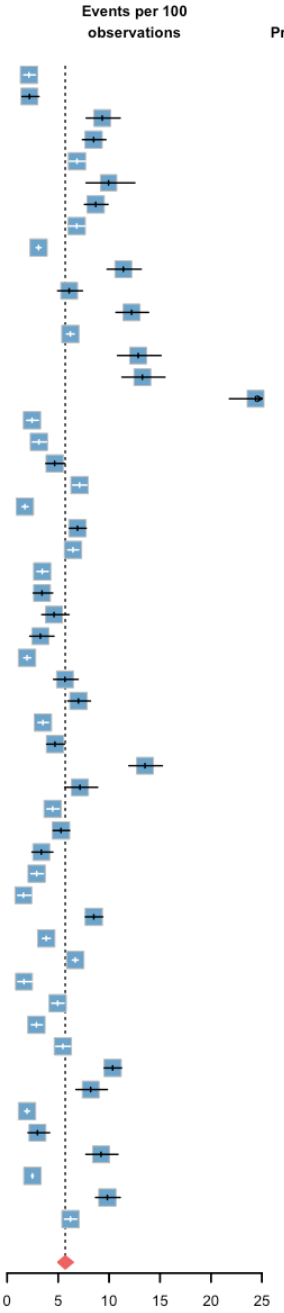

Figure S3. Pooled prevalence of peripheral artery disease in China (N=54)

Note: CI, confidence interval.

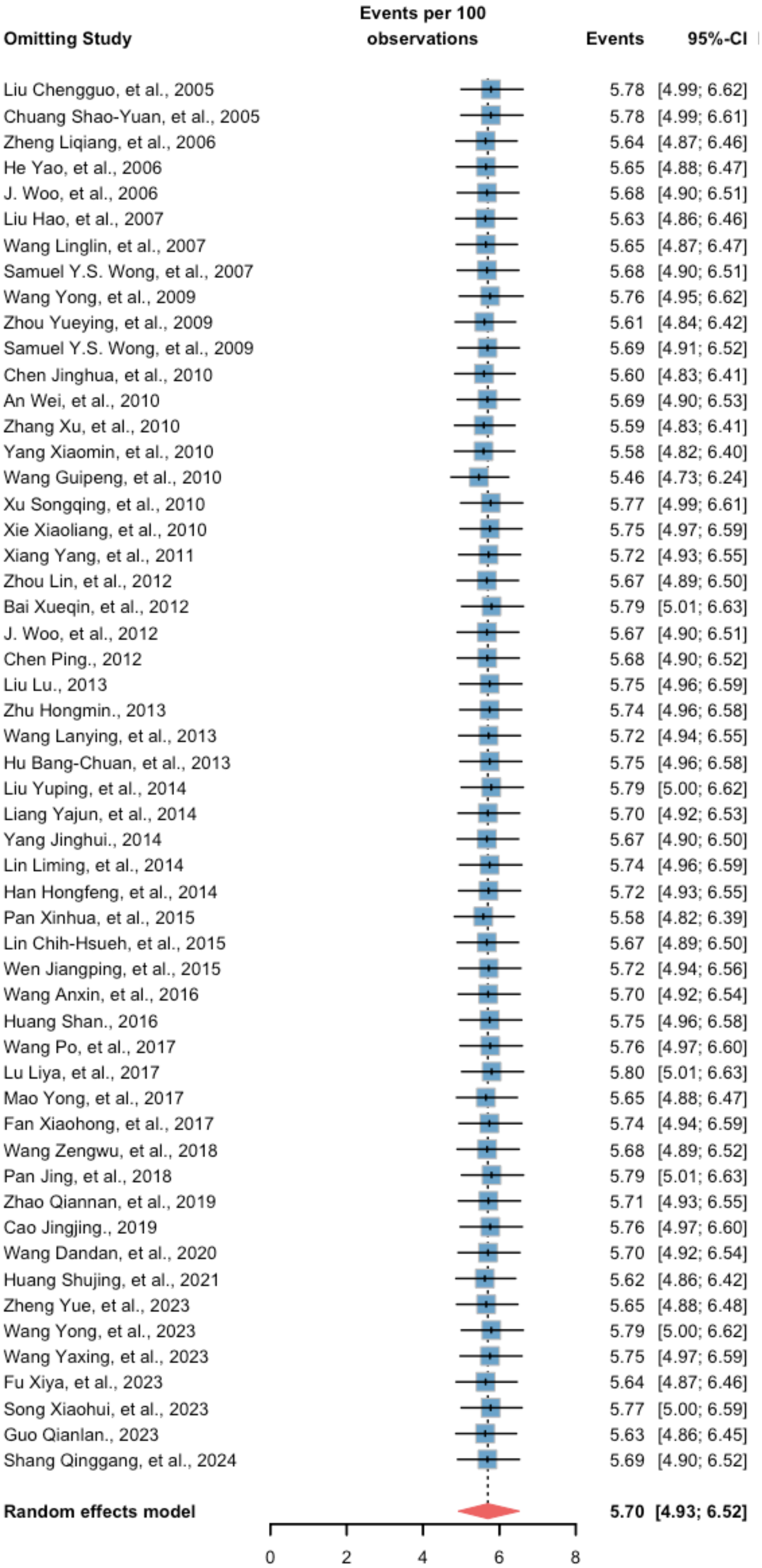

Figure S4. Leave-one-out sensitivity analysis of peripheral artery disease prevalence in China (N=54)

Note: CI, confidence interval; I^2, I-square.

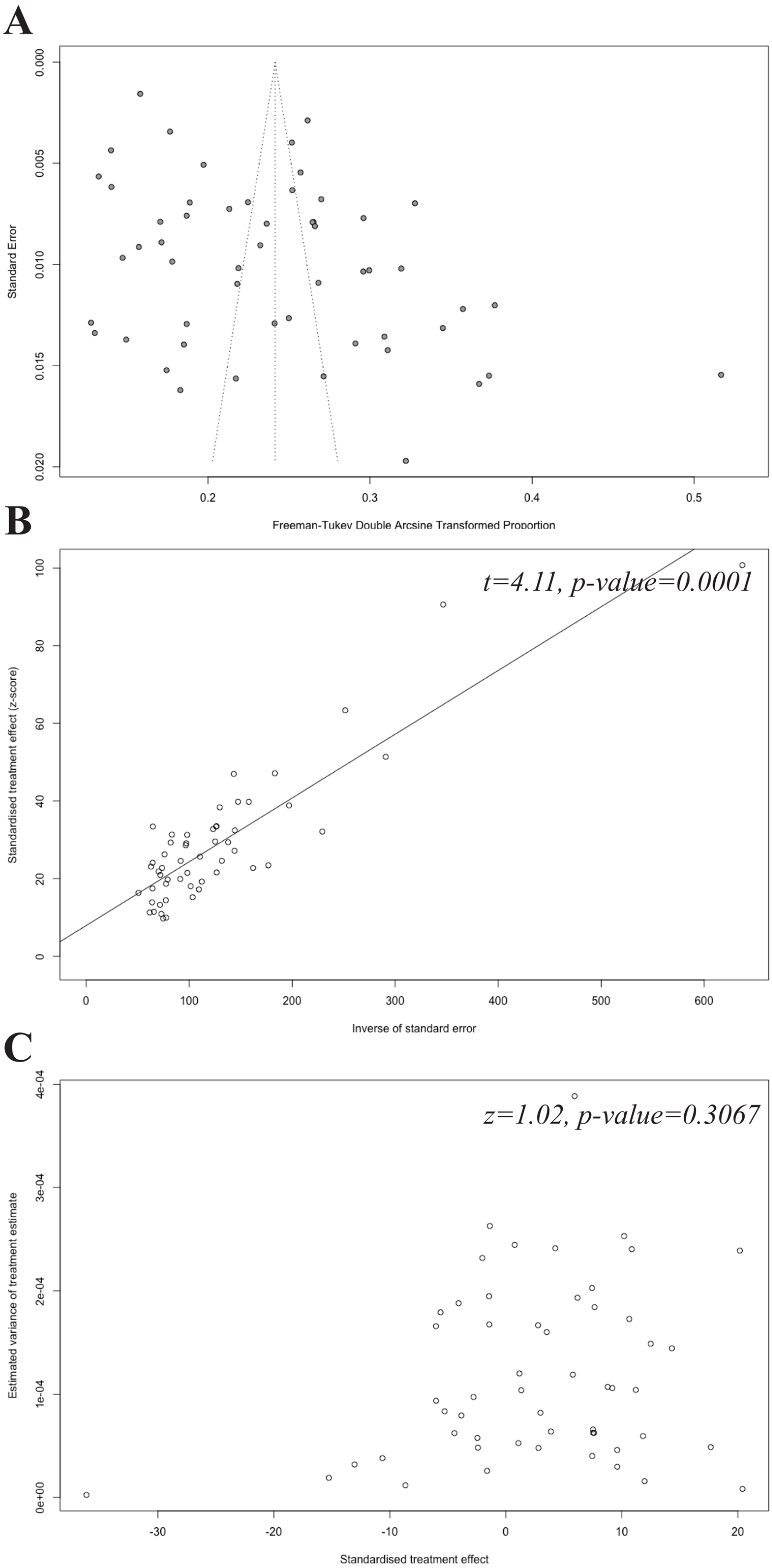

Figure S5. Publication bias in studies on peripheral artery disease prevalence in China (N=54)

Note: ***Panel A.*** *Funnel plot;* ***Panel B.*** *Egger's test;* ***Panel C.*** *Begg's test.*

Section 6. Articles retained for analysis (N=54)

Article ID	Reference
**P-01**	Liu Chengguo, Ruan Liansheng. 刘成国,阮连生. Prevalence of peripheral arterial disease and its risk factors in Zhoushan fishery area in Zhejiang Province （浙江省舟山渔区外周动脉病患病率调查）[J]. Chinese Journal of Geriatrics (R7 中华老年医学杂志). 2005. 24(11): 863-865.
**P-02**	Chuang Shao-Yuan, Chen Chen-Huan, Cheng Chien-Ming, et al. Combined use of brachial-ankle pulse wave velocity and ankle-brachial index for fast assessment of arteriosclerosis and atherosclerosis in a community [J]. International Journal of Cardiology. 2004,98(2005): 99–105.
**P-03**	Zheng Liqiang, Yu Jinming, Li Jue, et al. 郑黎强,余金明,李觉,等. Mean level of ankle brachial index and rate of peripheral arterial disease among people with different levels of body mass index in Yanbian, Sichuan （四川盐边县不同体质指数人群的踝臂指数及下肢外周动脉病患病率调查）[J]. Chinese Journal of Arteriosclerosis (中国动脉硬化杂志). 2006. 14(8): 717-720.
**P-04**	He Yao, Jiang Yong, Wang Jie, et al. Prevalence of peripheral arterial disease and its association with smoking in a population-based study in Beijing, China [J]. Journal of Vascular Surgery. 2006,44(2): 333-338.
**P-05**	J. Woo, H. Lynn, S.Y.S. Wong, et al. Correlates for a low ankle–brachial index in elderly Chinese [J]. Atherosclerosis. 2006,186(2): 360-366.
**P-06**	Liu Hao, Yu Jinming, Li Jue, et al. 刘浩,余金明,李觉,等. The relationship between high-sensitivity C-reactive protein and peripheral artery disease （高敏感反应蛋白与外周动脉疾病的关系）[J]. Chinese Journal of Internal Medicine (中华内科杂志). 2007. 46(5): 373-375.
**P-07**	Wang Linglin, Shi Rongkang, Wu Jun, et al. 王玲琳,施荣康,吴军,等. Study on the prevalence of peripheral artery disease in an urban population aged 60 years and over in Chengqiao community, Shanghai （上海市程桥社区老年人外周动脉疾病患病率的调查）[J]. Chinese Journal of Geriatrics (中华老年医学杂志). 2007. 26(7): 533-535.
**P-08**	Samuel Y.S. Wong, Jean Woo, Athena W.L. Hong, et al. Clinically relevant depressive symptoms and peripheral arterial disease in elderly men and women: results from a large cohort study in Southern China [J]. Journal of Psychosomatic Research. 2007,63(5): 471-476.
**P-09**	Wang Yong, Li Jue, Xu Yawei, et al. 王勇,李觉,徐亚伟,等. Prevalenceofperipheral arterial disease and correlative risk factorsamong naturalpopulation in China （中国自然人群下肢外周动脉疾病患病率及相关危险因素）[J]. Chinese Journal of Cardiology (中华心血管病杂志). 2009, 29(12): 1127-1131.
**P-10**	Zhou Yueying, Chen Jinghua, Zhang Zhihong. 周月英,陈晶华,张志宏. Prevalence of lower extremity arterial disease in 1447 old residents in Rong-gui community （1447 例容桂社区老年人下肢动脉疾病的流行病学调查）[J]. Chinese Journal of Geriatric Heart Brain and Vessel Diseases (中华老年心脑血管病杂志). 2009. 11(9): 705-708.
**P-11**	Samuel Y.S. Wong, Jason Chi Shun Leung, Jean Woo Sexual activity, erectile dysfunction and their correlates among 1,566 older Chinese men in Southern China [J]. International Society for Sexual Medicine. 2009,6(1): 74-80.
**P-12**	Chen Jinghua, Zhou Yueying, Li Siqiao, et al. 陈晶华,周月英,李思乔,等. Investigation of lower extremity arterial disease and its community care intervention in old residents （老年人群下肢动脉疾病调查及社区护理干预）[J]. Chinese General Practice (中国全科医学). 2010. 10(4A): 1112-1114.
**P-13**	An Wei, Li Xian, Wang Xin, et al. 安伟,李贤,王馨,等. Hypertension and peripheral arterial diseases （高血压与外周动脉疾病的关系）[J]. Journal of Peking University (Health Sciences) (北京大学学报（医学版）). 2010. 42(6): 667-670.

Article ID	Reference
**P-14**	Zhang Xu, Sun Zhenxue, Di Rujie. et al. 张旭,孙振学,邸茹杰,等. Evalution of ankle brachial index on elderly population with peripheral artery disease （踝臂指数评价老年外周动脉疾病的价值）[J]. Chinese Journal of Practical Internal Medicine (中国实用内科杂志). 2010,30(S1):15-17.
**P-15**	Yang Xiaomin, Sun Gang, Ding Yancheng, et al. 杨晓敏,孙刚,丁燕程,等. Association between lower ankle brachial index and cardiovascular risk factors in middle-old aged people （中老年人群踝臂指数减低的相关因素研究）[J]. Molecular Cardiology of China (中国分子心脏病学杂志). 2010,10(2): 98-101.
**P-16**	Wang Guipeng, Cui Fengqin, Zhang Xiangyang. 王贵鹏,崔凤琴,张向阳. Prevalence survey and analysis of correlative risk factors of arteriosclerosis obliterans of lower limbs in aged patients from community （社区老年人下肢动脉硬化闭塞症患病率调查及相关危险因素分析）[J]. Endemic Diseases Bulletin (China) (地方病通报). 2010,25(1): 14-17.
**P-17**	Xu Songqing, Cao Huie, Jin Zhong Qiang, et al. 许松青,曹惠娥,金忠强,等. Ankle-brachial index survey of 2994 workers*（2994 名职工踝臂指数调查）[J]. Zhejiang Preventive Medicine (浙江预防医学). 2010,22(11): 69-70.
**P-18**	Xie Xiaoliang, Xing Yan, Li Jue, et al. 谢晓亮,邢燕,李觉,等. A survey of ankle-brachial index among natural population in Shanghai and Inner Mongolia Autonomous Region （上海与内蒙古自治区两地自然人群踝臂指数的调查研究）[J]. Chinese Journal of Internal Medicine (中华内科杂志). 2012,51(10): 774-776.
**P-19**	Xiang Yang, Peng Xiao, Ma Yitong, et al. 向阳,彭潇,马依彤,等. Prevalence of peripheral arterial disease and correlative risk factors in Hazakh residents from Fuhai area of Xinjiang Aletai （新疆阿勒泰福海地区哈萨克族居民 PAD 患病率及危险因素分析）[J]. Journal of Xinjiang Medical University (新疆医科大学学报). 2011,34(2): 115-119.
**P-20**	Zhou Lin, Zhang Jie, Xu Yu, et al. 周琳,张婕,徐瑜,等. The association between peripheral arterial disease and body mass index in a community-based population （社区人群中外周动脉病与体质量指数相关性的研究）[J]. Journal of Internal Medicine Concepts & Practice (内科理论与实践). 2012,7(4): 289-292.
**P-21**	Bai Xueqin, Xie Yan, Zhao Runshuan, et al. 白雪琴,谢燕,赵润栓,等. Effects of metabolic syndrome and its components on ankle-brachial index （代谢综合征及其组分对踝臂指数的影响）[J]. Chinese Journal of Convalescent Medicine (中国疗养医学). 2012,21(11): 976-978.
**P-22**	J. Woo, N.L.S. TANG, J. LEUNG, et al. The Alu polymorphism of angiotensin I converting enzyme (ACE) and atherosclerosis, incident chronic diseases and mortality in an elderly Chinese population [J]. The Journal of Nutrition, Health & Aging. 2012,16(3): 262-268.
**P-23**	Chen Ping. 陈平. Prevalence of peripheral arterial disease and correlative risk factors in Xinjiang Uygur and Kazak adult population （新疆维吾尔族、哈萨克族外动脉疾病患病率及危险因素分析）[D]. Xinjiang Medical University (新疆医科大学). 2012.
**P-24**	Liu Lu. 刘璐. Prevalence of metabolic syndrome and peripheral artery disease in a natural population aged 40 years and above in Guiyang City （贵阳市 40 岁及以上自然人群代谢综合征并外周动脉疾病的患病率调查）[D]. Guiyang Medical University (贵阳医学院). 2013
**P-25**	Zhu Hongmin. 朱虹岷. Reference value of bachial-ankle pulse wave velocity and its determinants among community-dwelling elderly participants （上海社区老年人群臂踝脉搏波传导速度与踝臂指数的调查研究）[D]. Fudan University (复旦大学). 2013.
**P-26**	Wang Lanying, Ren Shuhong, Luo Bingquan. 王岚英,任淑红,骆秉铨. An epidemiological survey of peripheral vascular disease of lower extremity in community*（社区人群下肢外周血管病流行病学调查）[J]. Chinese Circulation Journal (中国循环杂志). 2013,8(S1): 18-19.

Article ID	Reference
**P-27**	Hu Bang-Chuan, Li Yan, Liu Ming, et al. Ankle brachial index in relation to the natriuretic peptide system polymorphisms and urinary sodium excretion in Chinese [J]. Atherosclerosis. 2013,230(1): 86-91.
**P-28**	Liu Yuping, Shuai Ping, Wang Lin, et al. 刘玉萍,帅平,王林,等. Prevalence of lower extremity arterial disease and its risk factors in middle-aged and elderly population on health check-up （中老年健康体检者下肢外周动脉疾病患病情况及危险因素分析）[J]. Chinese Journal of Geriatrics (中华老年医学杂志). 2014,33(11): 1231-1234.
**P-29**	Liang Yajun, Yan Zhongrui, Sun Binglun, et al. Cardiovascular risk factor profiles for peripheral artery disease and carotid atherosclerosis among Chinese older people: a population-based study [J]. PLOS ONE. 2013,9(1): e85927.
**P-30**	Yang Jinghui. 杨景慧. Genetic association study with metabolic diseases and peripheral artery occlusive disease in elderly （老年人代谢性疾病及周围动脉闭塞性疾病基因关联研究）[D]. PLA Medical College (中国人民解放军医学院). 2014.
**P-31**	Lin Liming, Wu Shouling. 林黎明,吴寿岭. Correlation between fasting blood glucose and ankle-brachial index*（空腹血糖与踝臂指数的相关性）[J]. Chinese Journal of Gerontology (中国老年学杂志). 2014,34(9): 5549-5551.
**P-32**	Han Hongfeng, Li Yuqing, Wu Shouling. 韩红锋,李玉卿,吴寿岭. Distribution of ankle-brachial index in different sex populations*（不同性别人群踝臂指数的分布情况）[J]. Medical Information (医学信息). 2014,27(9): 156.
**P-33**	Pan Xinhua, Chen Gencheng, Zheng Canjie, et al. 潘新花,陈根成,郑灿杰,等. Survey of residents' ankle-brachial index level*（居民踝臂指数水平调查）[J]. Zhejiang Preventive Medicine (浙江预防医学). 2014,26(1): 67-68.
**P-34**	Lin Chih-Hsueh, Chou Che-Yi, Liu Chiu-Shong, et al. Association between frailty and subclinical peripheral vascular disease in a community-dwelling geriatric population: Taichung Community Health Study for Elders [J]. Geriatrics & Gerontology International. 2015,15(3): 261-267.
**P-35**	Wen Jiangping, Yang Jingang, Shi Yujie, et al. Comparisons of different metabolic syndrome definitions and associations with coronary heart disease, stroke, and peripheral arterial disease in a rural Chinese population [J]. PLOS ONE. 2015,10(5): e0126832.
**P-36**	Wang Anxin, Jiang Ruixuan, Su Zhaoping, et al. A low ankle-brachial index is associated with cognitive impairment: The APAC study [J]. Atherosclerosis. 2016,255: 90-95.
**P-37**	Huang Shan. 黄姗. The epidemiolgical investigation of ankle brachial index and the risk factors which affect Yongfu and Liujiang rural residents （永福县和柳江县农村居民踝臂指数及其相关危险因素调查）[D]. Guangxi Medical University (广西医科大学). 2016.
**P-38**	Wang Po, Du Rui, Lin Lin, et al. Association between Free Triiodothyronine Levels and Peripheral Arterial Disease in Euthyroid Participants [J]. Biomedical and environmental sciences: BES. 2017. 30(2): p. 128-133.
**P-39**	Lu Liya, Jiang Chaoqiang, Danny F Mackay, et al. Exposure to second hand smoke and risk of peripheral arterial disease in southern Chinese non-smokers: The Guangzhou Biobank Cohort Study-Cardiovascular Disease Sub-cohort [J]. Vascular. 2017. 25(3): p. 283-289.
**P-40**	Mao Yong, Huang Yixiang, Yu Haining, et al. Incidence of Peripheral Arterial Disease and Its Association with Pulse Pressure: A Prospective Cohort Study [J]. Frontiers in Endocrinology (Lausanne). 2017. 8: p. 333.
**P-41**	Fan Xiaohong, Sahir Kalim, Ye Wenling, et al. Urinary Stone Disease and Cardiovascular Disease Risk in a Rural Chinese Population [J]. Kidney International Reports. 2017. 2(6): p. 1042-1049.

Article ID	Reference
**P-42**	Wang Zengwu, Wang Xin, Hao Guang, et al. A national study of the prevalence and risk factors associated with peripheral arterial disease from China: The China Hypertension Survey, 2012–2015 [J]. International Journal of Cardiology. 2018.
**P-43**	Pan Jing, Xu Lin, Tai Hing Lam, et al. Relationship between pulmonary function and peripheral vascular function in older Chinese: Guangzhou biobank cohort study-CVD [J]. BMC Pulmonary Medicine. 2018. 18(1): p. 74.
**P-44**	Zhao Qiannan, Wang Chunxiu, Guan Shaochen, et al. 赵倩南,王淳秀,关绍晨,等. Prevalence characters of peripheral artery disease and associated factors among Beijing residents aged equal and above 35 years old （北京地区 35 岁及以上人群外周动脉疾病患病率特点及影响因素分析）[J]. Chinese Journal of Cardiology (中华心血管病杂志). 2019. 47(12): p. 1000-1004.
**P-45**	Cao Jingjing. 曹婧婧. Relationship of serum fasting glucose with arterial stiffness and peripheral arterial disease among hypertensive population in rural areas of two cities （两城市农村高血压人群空腹血糖与动脉硬化及外周动脉疾病的关联性分析）[D]. Anhui Medical University (安徽医科大学). 2019.
**P-46**	Wang Dandan, Zhang Qian, Wang Anxin, et al. Ideal Cardiovascular Health Metrics on the New Occurrence of Peripheral Artery Disease: A Prospective Cohort Study in Northern China [J]. Scientific Reports. 2020. 10(1): p. 9660.
**P-47**	Huang Shujing, Sun Hongyan, Yu Jia, et al. The Interaction Between Self-Reported Sleep Duration and Physical Activity on Peripheral Artery Disease in Chinese Adults: A Cross-Sectional Analysis in the Tianning Cohort Study [J]. Risk Management and Healthcare Policy. 2021. 14: p. 4063-4072.
**P-48**	Zheng Yue, Sun Yuechao, Zhang Ziwei, et al. Combined Effect of Dynapenia and Abdominal Obesity on the Prevalence of Peripheral Artery Disease in Older Adults Over 75 Years Old in China [J]. Clinical and Applied Thrombosis/Hemostasis. 2023. 29: p. 10760296231169503.
**P-49**	Wang Yong, Guo Xiaoyan, Zhang Yi, et al. Different associations of general and abdominal obesity with upper and lower extremity artery disease among a community population in China [J]. Nutrition & Metabolism. 2023. 20(1): p. 14.
**P-50**	Wang Yaxing, Wang Qian, RAHUL A. JONAS, et al. Prevalence and Associations of Peripheral Arterial Disease in China. The Beijing Eye Study [J]. American journal of ophthalmology. 2023.
**P-51**	Fu Xiya, Qi Yiqiong, Han Peipei, et al. Relationship Between Physical Performance and Peripheral Arterial Diseases in Different Age Groups of Chinese Community-Dwelling Older Adults [J]. Journal of Atherosclerosis and Thrombosis. 2023. 30(7): p. 778-785.
**P-52**	Song Xiaohui, Liu Bo, Lei Fang, et al. The Association Between Metabolic Dysfunction-Associated Fatty Liver Disease and Peripheral Arterial Disease in the Chinese Population [J]. Diabetes, Metabolic Syndrome and Obesity. 2023. 16: p. 373-384.
**P-53**	Guo Qianlan. 郭倩岚. Association of serun uric acid level and peripheral arterial disease (PAD) in women over 35 years old, in Changzhou （常州市 35 岁以上女性血清尿酸水平与下肢动脉硬化闭塞症(PAD)的关联性研究）[D]. Soochow University (苏州大学). 2020.
**P-54**	Shang Qinggang, Lv Deliang, Xie Wei, et al. 尚庆刚,吕德良,谢尉,等. The prevalence of peripheral arterial disease and its relationship with obesity status among residents in Shenzhen City（深圳市居民外周动脉疾病患病现况及与肥胖状态关系研究）[J]. China Tropical Medicine (中国热带医学). 2024. 24(6).

Note: The Chinese publication list employed the journals' official English names or abbreviations, English titles were obtained from journals or literature databases (CNKI, Wanfang and VIP). Where official English translation of journal names is not available, a pinyin title is adopted; where the English translation of titles is not available, I translated the titles, labelled with "" and marked as green.*
